# Evaluation of Digital Interventions for Physical Activity Promotion: Scoping Review

**DOI:** 10.2196/37820

**Published:** 2022-05-23

**Authors:** Karina Karolina De Santis, Tina Jahnel, Katja Matthias, Lea Mergenthal, Hatem Al Khayyal, Hajo Zeeb

**Affiliations:** 1 Department of Prevention and Evaluation Leibniz Institute for Prevention Research and Epidemiology- BIPS Bremen Germany; 2 Leibniz-Science Campus Digital Public Health Bremen Bremen Germany; 3 Faculty 11 Human and Health Sciences University of Bremen Bremen Germany; 4 Faculty of Electrical Engineering and Computer Science University of Applied Science Stralsund Stralsund Germany; 5 Faculty of Engineering and Mathematics Bielefeld University of Applied Science Bielefeld Germany

**Keywords:** evaluation, digital interventions, physical activity, scoping review, review, health promotion, behavior change theory, digital health, public health, digital technology

## Abstract

**Background:**

Digital interventions are interventions supported by digital tools or technologies, such as mobile apps, wearables, or web-based software. Digital interventions in the context of public health are specifically designed to promote and improve health. Recent reviews have shown that many digital interventions target physical activity promotion; however, it is unclear how such digital interventions are evaluated.

**Objective:**

We aimed to investigate evaluation strategies in the context of digital interventions for physical activity promotion using a scoping review of published reviews. We focused on the target (ie, user outcomes or tool performance), methods (ie, tool data or self-reported data), and theoretical frameworks of the evaluation strategies.

**Methods:**

A protocol for this study was preregistered and published. From among 300 reviews published up to March 19, 2021 in Medline, PsycINFO, and CINAHL databases, 40 reviews (1 rapid, 9 scoping, and 30 systematic) were included in this scoping review. Two authors independently performed study selection and data coding. Consensus was reached by discussion. If applicable, data were coded quantitatively into predefined categories or qualitatively using definitions or author statements from the included reviews. Data were analyzed using either descriptive statistics, for quantitative data (relative frequencies out of all studies), or narrative synthesis focusing on common themes, for qualitative data.

**Results:**

Most reviews that were included in our scoping review were published in the period from 2019 to 2021 and originated from Europe or Australia. Most primary studies cited in the reviews included adult populations in clinical or nonclinical settings, and focused on mobile apps or wearables for physical activity promotion. The evaluation target was a user outcome (efficacy, acceptability, usability, feasibility, or engagement) in 38 of the 40 reviews or tool performance in 24 of the 40 reviews. Evaluation methods relied upon objective tool data (in 35/40 reviews) or other data from self-reports or assessments (in 28/40 reviews). Evaluation frameworks based on behavior change theory, including goal setting, self-monitoring, feedback on behavior, and educational or motivational content, were mentioned in 22 out of 40 reviews. Behavior change theory was included in the development phases of digital interventions according to the findings of 20 out of 22 reviews.

**Conclusions:**

The evaluation of digital interventions is a high priority according to the reviews included in this scoping review. Evaluations of digital interventions, including mobile apps or wearables for physical activity promotion, typically target user outcomes and rely upon objective tool data. Behavior change theory may provide useful guidance not only for development of digital interventions but also for the evaluation of user outcomes in the context of physical activity promotion. Future research should investigate factors that could improve the efficacy of digital interventions and the standardization of terminology and reporting in this field.

**International Registered Report Identifier (IRRID):**

RR2-10.2196/35332

## Introduction

The field of digital public health aims to promote and improve the health of people and communities through the application of digital technologies [1,2]. Digital technologies specifically designed to promote and improve health have emerged on a large scale and already permeate seemingly all aspects of daily life. For example, interventions supported by digital technologies (ie, digital interventions) are becoming increasingly popular in the context of healthy lifestyles and behavior change, including physical activity promotion [3]. Given the rapid growth in the number and sophistication of digital technologies, the use of mobile wearable devices or smartphone apps has been found to be a scalable and cost-effective way of promoting physical activity–related behavior change [4].

Digital technologies have tremendous potential to be incorporated into health interventions that are grounded in behavioral theory. Such digital interventions can include a variety of potentially useful behavior change techniques and can be tailored to meet the needs of individuals or populations [5]. Behavior change theory refers to the active ingredients of any given intervention that aim to evoke a change in behavior (eg, increase physical activity), which have been classified according to their nature [6,7]. Various components of behavior change theory have been used in digital interventions for physical activity promotion, including goal setting, activity monitoring with feedback, and shaping knowledge [8,9]. In particular, the use of goal setting, social incentives, and graded tasks may improve the physical activity outcomes of digital interventions [10].

Little is known about how digital interventions help shape behavior in real-world settings. This suggests there is a need to evaluate and understand factors related to intervention success or failure [11,12]. Success or failure depends on the context of use, including structural issues in the environment in which an intervention is used, available infrastructure, the health needs that are being addressed, and the ease of use of the technology [3,13]. Thus, an evaluation of novel digital interventions is important, not only in terms of efficacy but also, to justify and inform policy, program, and funding decisions. In Germany, digital health apps that are used as medical devices must undergo an evaluation process similar to that undergone by other medical procedures, while other digital interventions with a primary focus on prevention, such as digital interventions for physical activity promotion, are not required to undergo such an evaluation process [14]. More importantly, when evaluations are omitted, it becomes the user’s responsibility to identify which digital interventions may be effective and useful, and consequently, users bear the risk of using ineffective, or even potentially harmful, solutions.

One key issue in this area of research is the lack of frameworks or guidelines specifically addressing the evaluation of digital interventions. Although assessment criteria for health-related technologies in general have been developed previously, their focus is generally neither on digital technologies [15] nor on a public health context [16]. Health technology assessment, for example, is a methodology for the systematic and transparent evaluation of medical procedures and technologies under medical, economic, social, ethical, and economic aspects with the aim of supporting associated decision-making processes [17]. While health technology assessment is not specifically designed for digital interventions, various organizations that are engaged in health technology assessment were involved in creating or guiding the development of standards for the evidence required for digital interventions. For example, the National Institute for Health and Care Excellence recently developed an Evidence Standards Framework for Digital Health Technologies for assessing the effectiveness and cost-effectiveness of digital interventions within the UK health care system [18]. Currently, however, health technology assessment frameworks such as this [18] mainly focus on evaluating the clinical rather than the public health outcomes of novel digital interventions.

We initially planned to conduct a scoping review in two phases: (1) scoping review of existing reviews (ie, review of reviews) and (2) scoping review of primary studies [19]. As explained in a subsequent study protocol [20], this scoping review addresses phase 1 of the study. Phase 2 will depend on the outcomes of phase 1 of the study; specifically, phase 1 of the study will provide evidence to support a decision for or against conducting a new scoping review of primary literature. Such a decision needs to be evidence-based to prevent any research waste that occurs when new reviews are conducted despite the existence of other reviews that address the same aims.

**Figure 1:**
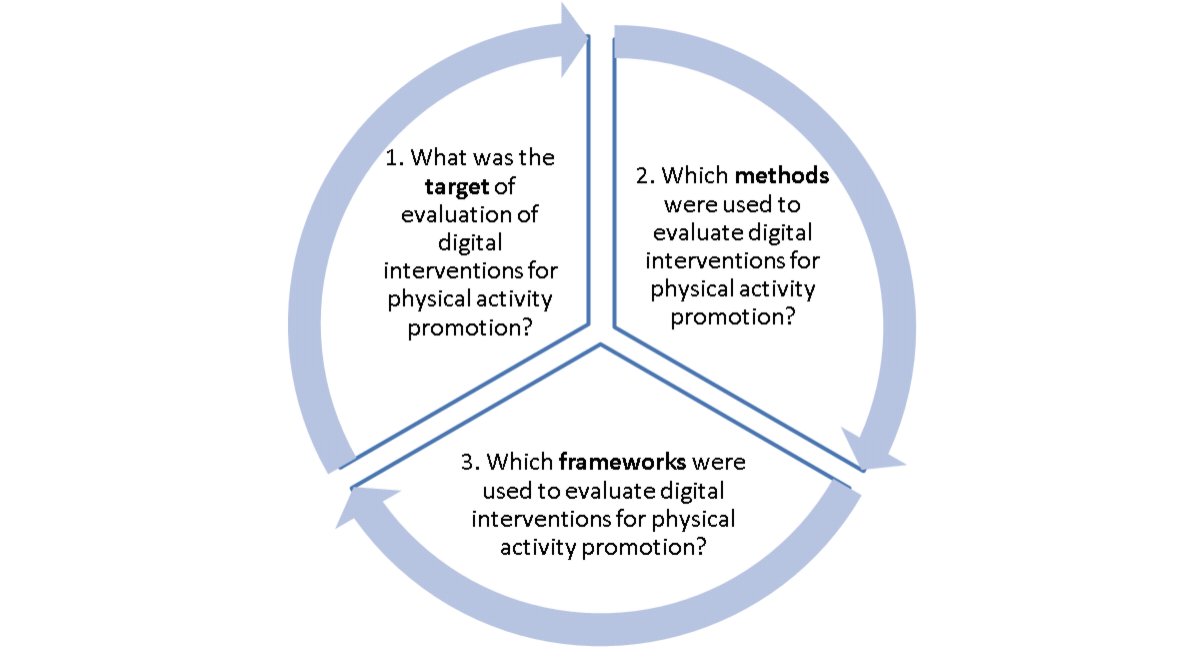
Objectives of this scoping review.

The aim of this scoping review was to investigate the evaluation strategies in the context of digital interventions for physical activity promotion that were addressed in other published reviews. The 3 main objectives of this scoping review address the target (ie, user outcomes or tool performance), methods (ie, tool data or self-reported data), and theoretical frameworks of such evaluations (Figure 1). In addition, we also aim to summarize the evidence gaps identified in other published reviews.

## Methods

### Study Design

This study was a scoping review and adheres to PRISMA-ScR (Preferred Reporting Items for Systematic Reviews and Meta-analyses Extension for Scoping Reviews) guidelines [21]. The PRISMA-ScR checklist is reported in Table S1 in Multimedia Appendix 1.

### Protocol and Registration

The protocol for this scoping review was prospectively registered [19] and published [20]. We chose a scoping review methodology because our objectives focus broadly on the strategies required to evaluate digital interventions rather than the efficacy of digital interventions (which is typically the case in overviews). We apply 2 aspects taken from the overview methodology: (1) we assessed the overlap among primary studies cited in the included reviews to investigate the uniqueness of existing evidence, and (2) we appraised the included systematic reviews to investigate the sources of weaknesses in existing evidence. Since the methods applied in this scoping review were already reported in detail in our published protocol [20], only a short summary is provided here. There were no changes between the published protocol [20] and the objectives, methods, and results reported in this scoping review.

### Eligibility Criteria

The eligibility criteria ([20], Textbox 1) for this scoping review were derived from the Population, Intervention, Comparison, Outcome, and Study type (PICOS) criteria.

**Inclusion criteria for this scoping review.**

**Population**

- Any health status (healthy or clinical human samples)
- Any age (children or adults)

**Intervention**

- Digital interventions for physical activity promotion

**Comparison**

- Any other intervention or no intervention

**Outcome**

- Evaluation of any outcome in the context of physical activity promotion

**Study type**

- Any review (systematic, scoping, rapid, narrative, overview)
- Papers published in peer-reviewed journals, in English or German, available in full-text

### Information Sources

We used (1) international databases (MEDLINE, PsycINFO, and CINAHL) and (2) the reference sections of studies (reviews) included in our scoping review.

### Search

The electronic search strategy (Multimedia Appendix 2) was developed and performed under the supervision of an experienced librarian. The electronic search was performed from database inception to March 19, 2021, without any limits, in 3 international databases.

### Selection of Sources of Evidence

The electronic search returned 8272 records that were stored and processed in EndNote X9 (Clarivate); after duplicates were removed, 4912 records remained. Reviews of any type were identified using smart group settings in EndNote and assessed for eligibility by any 2 authors independently. Based on title and abstract screening, reviews that met the inclusion criteria were selected for full-text screening. The reference sections of eligible full-text reviews were also manually screened to identify additional relevant reviews. Final eligibility was decided by consensus. Final list of included and excluded studies is shown in Table S2 in Multimedia Appendix 1).

### Data Charting

Data were coded into a self-developed spreadsheet (Excel, version 10; Microsoft Inc). The spreadsheet was pilot-tested and calibrated within the team. Data coding was performed independently by 2 authors, and consensus was reached by discussion.

### Data Items

Data items (Textbox 2) were coded either quantitatively into predefined categories or qualitatively using definitions or author statements from the included reviews.

The operational definitions of the 2 key concepts (digital interventions and physical activity promotion) are summarized in Textbox 3.

**Data items in this scoping review.**

1. Bibliographic information (publication year, author region, conflict of interest)
2. Population details (health status and age)
3. Digital intervention details
4. Comparison condition
5. Outcome in the context of physical activity promotion
6. Study details
  - Review type
  - Primary studies in review (number, designs, overlap among primary studies cited in reviews)
7. Evaluation strategy details (target, methods, theoretical frameworks)
8. Evidence gaps (requirements for efficacy and ideas for future research)

**Operational definitions applied in this scoping review.**

**Digital intervention**

- Digital intervention was defined as any intervention delivered or supported by digital tools or digitally supported technologies for automated and continuous self-monitoring and feedback. This includes mobile apps, wearable activity trackers and web-based software but excludes pedometers and accelerometers that do not offer feedback throughout time [22]. Reviews were included if only a minority of their primary studies incorporated pedometers or accelerometers.

**Physical activity promotion**

- Physical activity promotion was defined as any primary outcome targeting general fitness or mobility. Reviews were excluded if physical activity promotion was assessed as part of healthy lifestyle, as a secondary outcome to management of weight or blood sugar, or as part of rehabilitation after sport injuries, surgeries, or in neurological disorders.

### Critical Appraisal of Individual Sources of Evidence

We performed critical appraisals using AMSTAR2 (A Measurement Tool to Assess Systematic Reviews, version 2) [23]) of all systematic reviews to identify weaknesses in existing evidence. The appraisal procedure was explained in detail in the published protocol [20]. Two authors appraised all systematic reviews independently and reached consensus by discussion. The overall confidence ratings in the results of each systematic review (high, moderate, low, or critically low) were established based on the type and the number of weaknesses in each review [23] (Multimedia Appendix 3).

### Synthesis of Results

Coded data were synthesized using either descriptive statistics of quantitative data (relative frequencies out of all studies) or narrative descriptions of qualitative data (by identifying common themes). The AMSTAR2 appraisal outcomes (overall confidence ratings) were synthesized for all systematic reviews using a bar graph. Evidence maps were used to visualize the results based on the objectives of this scoping review (Figure 1).

## Results

### Included Studies

#### Study Selection

Of 4912 records identified in our electronic search, 300 were designated as reviews of any type based on the titles or abstracts (Table S2 in Multimedia Appendix 1), and 40 reviews were found to meet eligibility criteria: 36/40 reviews from the electronic search and 4/40 reviews from the manual search of reference sections of these 36 reviews. The majority of the 40 included studies were systematic reviews, followed by scoping reviews; there was 1 rapid review (Table 1). All 40 reviews addressed the evaluation strategies for any outcome in the context of digital interventions for physical activity promotion in healthy or clinical samples. The digital interventions in all reviews were supported by digital tools, such as mobile phones, smartphone apps, wearable activity trackers, or the internet (ie, websites). The physical activity promotion outcomes in all reviews were general fitness or mobility measures (ie, steps per day, frequency of physical exercise at various intensities, meeting physical activity guidelines; Multimedia Appendix 4).

**Table 1:** A list of studies (40 reviews) included in this scoping review.

Study type	Studies (n=40)	Citation
Rapid review	1	[24]
Scoping review	9	[8,9,25-31]
Systematic review	30	[11,22,32-59]

#### Study Characteristics

**Figure 2:**
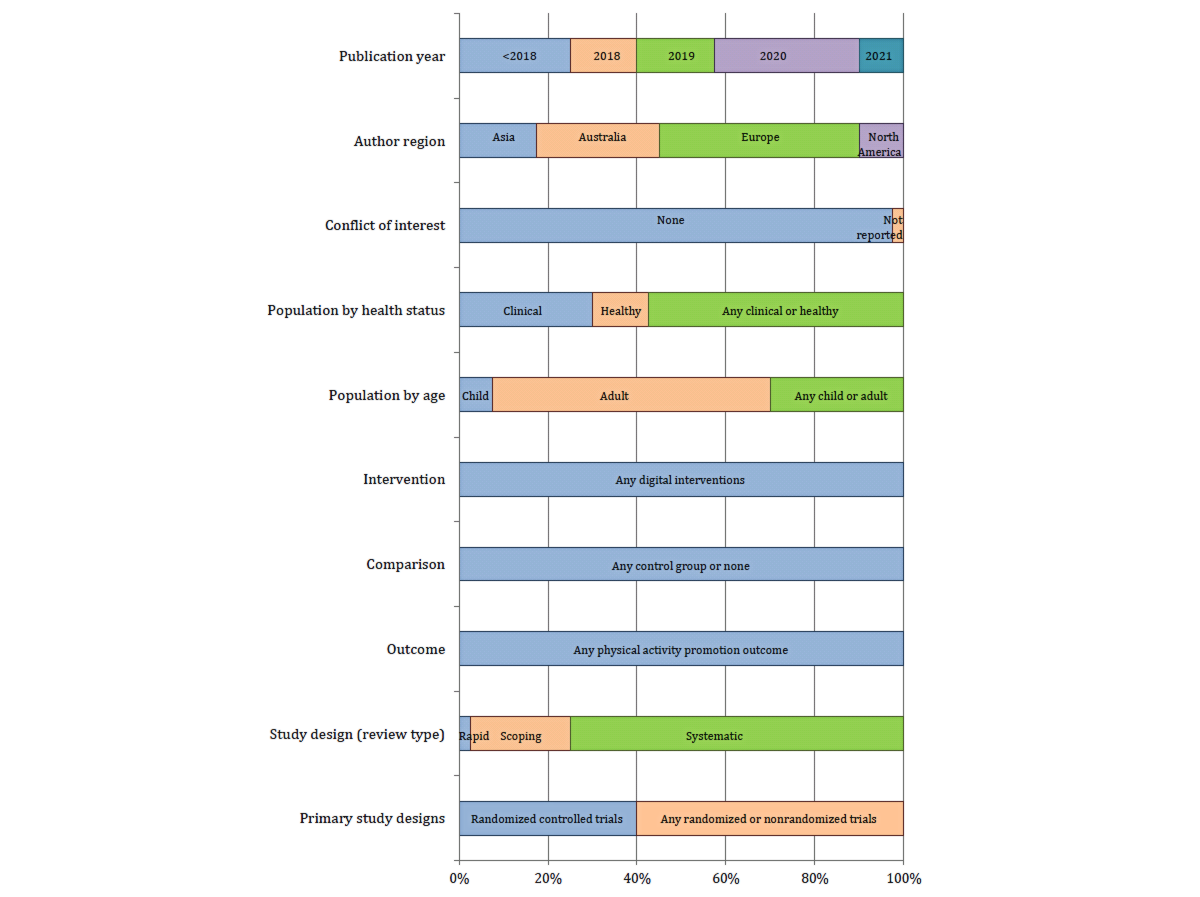
Study characteristics of 40 reviews.

Study characteristics of the individual reviews are shown in Figures S1 and S2 in Multimedia Appendix 1. Synthesis of study characteristics of all 40 reviews is shown in Figure 2. All 40 reviews were published in the period from 2007 to 2021. The majority were systematic reviews (30/40), published from 2019 to 2021 (24/40), originated from Europe (18/40) or Australia (11/40), and reported no conflicts of interest (39/40). All 40 reviews addressed any digital interventions for any physical activity promotion outcome relative to any control condition (other interventions or baseline physical activity). Most reviews included primary studies with any design (randomized controlled trials or non–randomized controlled trials: 24/40 reviews) adult populations (25/40 reviews), and any health setting (clinical or nonclinical: 23/40 reviews).

#### Overlap Among Primary Studies Cited in Reviews

The assessment of the overlap among primary studies showed that most primary studies were cited only once in any review (Textbox S1 in Multimedia Appendix 1). The 10 rapid or scoping reviews cited 278 unique published primary studies. Most of these studies (244/278, 87.8%) were cited only once in any review while the rest (34/278, 12.2%) were cited twice. The 30 systematic reviews cited 320 unique published primary studies. Most of these studies (249/320, 77.8%) were cited only once, others (67/320, 20.9%) were cited 2 to 4 times, and the minority (4/320, 1.2%) were cited either 5 times [60,61] or 6 times [62,63].

#### Quality Appraisal in Systematic Reviews

**Figure 3:**
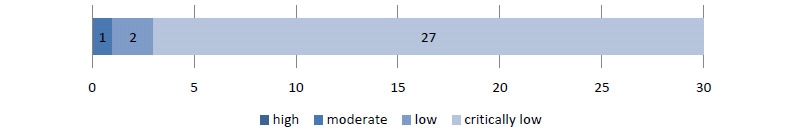
Overall confidence in the results of 30 systematic reviews.

The majority of systematic reviews (27/30, 90%) received critically low confidence ratings, and the remaining systematic reviews received either low (2/30, 6.7% [38,41]) or moderate (1/30, 3.3% [22]) confidence ratings (Figure 3). None of the systematic reviews received high confidence ratings. The 3 most common weaknesses among the 30 systematic reviews were that a list of excluded studies was not reported, a review protocol was not mentioned, and the sources of funding for the primary studies included in review were not reported.

### Evaluation of Digital Interventions for Physical Activity Promotion

#### Overall

**Figure 4:**
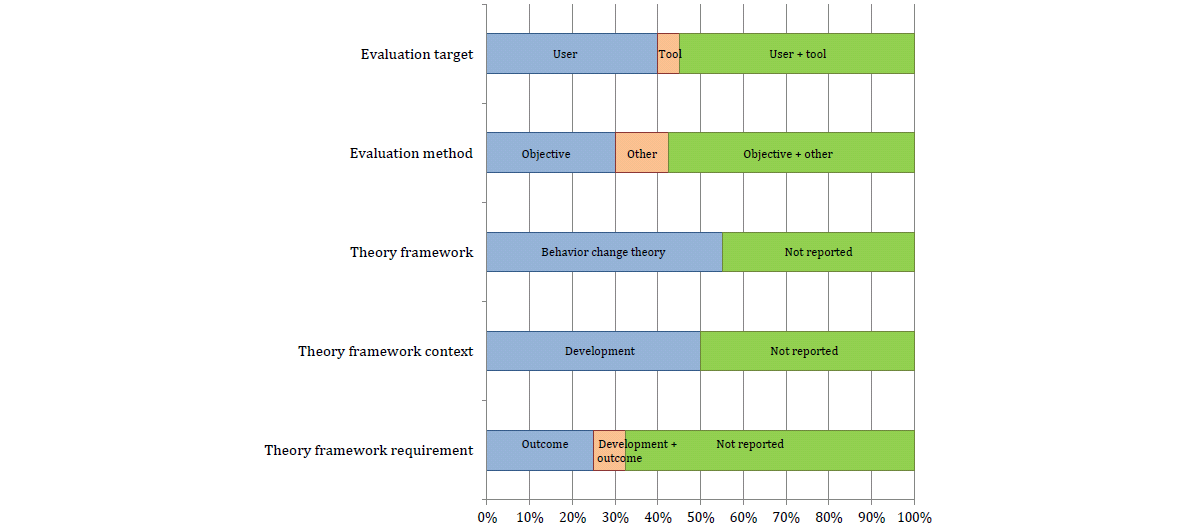
Evaluation strategies addressed in 40 reviews.

Evaluation strategies of digital interventions for physical activity promotion addressed in the individual reviews are shown in Figure S3 in Multimedia Appendix 1. The synthesis of evaluation strategies in all reviews (Figure 4) showed that, while all 40 reviews addressed evaluation targets and evaluation methods used to assess digital interventions for physical activity promotion, only just over half of reviews mentioned the evaluation frameworks.

#### Evaluation Targets

Evaluation target was either any user outcome (in 38/40 reviews) or tool performance (in 24/40 reviews). Both evaluation targets (user outcomes and tool performance) were mentioned in 22/40 reviews. User outcomes were described as efficacy, acceptability, usability, feasibility, or engagement with digital interventions for promoting physical activity while tool performance was typically mentioned in the context of tool validation (Multimedia Appendix 4).

#### Evaluation Methods

Evaluation methods relied upon either objective data (in 35/40 reviews) or other data (in 28/40 reviews). Both types of data (objective and other) were mentioned in 23/40 reviews. Objective data were automatic, tool-generated data, such as continuous recording of physical activity with wearable activity trackers or smartphone apps. Other data included self-reported data from questionnaires or rating scales (used to assess tool performance), tests, or assessments of user outcomes as well as various measures of engagement or coaching (Multimedia Appendix 4).

#### Evaluation Frameworks

Only just over half of all reviews (22/40) mentioned evaluation frameworks. All 22 reviews focused on various aspects of behavior change theory, such as goal setting, self-monitoring, feedback on behavior, and educational or motivational content. Among the 22 reviews, 20 discussed the context of evaluation frameworks and concluded that aspects of behavior change theory were predominantly used to develop digital interventions for physical activity promotion. Among all 40 reviews, 13 mentioned a need to incorporate evaluation frameworks to assess user outcomes of digital interventions in the context of physical activity promotion (Multimedia Appendix 4).

### Evidence Gaps

#### Overall

There were several evidence gaps identified in the 40 reviews (Table 2). The evidence gaps mentioned by the authors of the 40 reviews were synthesized with respect to two main themes: (1) requirements for efficacy and (2) ideas for future research.

**Table 2:** Evidence gaps in 40 reviews.

Review type, theme, and category				Studies, n
**Rapid and scoping reviews**				10
	**Requirements for efficacy**			
		Identify factors that could improve the effectiveness of digital interventions by increasing compliance and adherence to digital interventions (personalization, feedback, engagement with the tool, human support, and digital literacy)	7	
		Need for guidelines for evaluation and reporting / better reporting of digital interventions	3	
		Need for objective and homogeneous outcome measures required to evaluate digital interventions	3	
	**Ideas for future research**			
		Use and grounding of behavioral theory or include theoretical framework for digital interventions	3	
		Perform long-term studies / use longer follow-up for digital interventions	1	
**Systematic reviews**				30
	**Requirements for efficacy**			
		Identify factors that could improve the effectiveness of digital interventions by increasing compliance and adherence to digital interventions (personalization, feedback, engagement with the tool, human support, and digital literacy)	18	
		Need for objective and homogeneous outcome measures required to evaluate digital interventions	11	
		Need for guidelines for evaluation and reporting and better reporting of digital interventions	2	
	**Ideas for future research**			
		Perform long-term studies and use longer follow-up for digital interventions	13	
		Evaluation or better understanding of (clinical) effectiveness of digital interventions	9	
		Need for an appropriate study design in future studies (eg, high quality trials, rigorous study designs)	8	
		Use and grounding of behavioral theory or include theoretical framework for digital interventions	5	
		Investigation of cost-effectiveness of digital interventions	5	
		Inclusion of more diverse samples in the studies (eg, low-income countries, age groups)	3	

#### Requirements for Efficacy

Three main themes emerged in the context of assessing the efficacy of digital interventions for physical activity promotion. Most reviews mentioned the need to identify factors that could improve the effectiveness of digital interventions. The other themes were the need to objectively and homogeneously define the outcomes of digital interventions and the need for evaluation guidelines and better standardized reporting of digital interventions components and outcomes.

#### Ideas for Future Research

There were several ideas for future research. Two common themes among the reviews were a need for theoretical frameworks to evaluate digital interventions and a need for evaluation of digital interventions using studies with long-term follow-up. In addition, systematic reviews mentioned a need to understand the clinical effectiveness of digital interventions that should be studied using rigorous and high-quality study designs. Some systematic reviews also suggested investigating the cost-effectiveness of digital interventions and evaluating digital interventions in more diverse settings and samples, such as in samples with different sociodemographic characteristics.

## Discussion

### Principal Results

This scoping review shows that 40 reviews (rapid, scoping, or systematic) that had been published within the last 15 years mentioned the issue of evaluation of digital interventions for physical activity promotion. All reviews addressed different evaluation targets, which included user outcomes or tool performance in the context of physical activity promotion. The reviews mentioned that evaluation methods relied predominantly upon objective tool data, although data from self-reports or assessments were also used. Only approximately half of all reviews mentioned evaluation frameworks and concluded that various aspects of behavior change theory were applied to develop digital interventions but not to evaluate the user outcomes of such digital interventions.

### Interest in the Evaluation of Digital Interventions for Physical Activity Promotion in Past Reviews

We found that many reviews that have been published to date mentioned the issue of evaluation of digital interventions for physical activity promotion. While evaluation targets and methods were mentioned in all reviews, only some reviews addressed evaluation frameworks. Among these reviews, most suggest that evaluation frameworks seem to be considered in development of digital interventions; however, it is unclear if evaluation frameworks are used to evaluate the outcomes of digital interventions. There are several possible explanations for these findings First, most reviews aimed to synthesize the literature on the effects of digital interventions on various user outcomes, and information on evaluation frameworks may not have been coded from the primary studies by review authors. This seems unlikely because evaluation was often discussed in reviews meaning that details on evaluation frameworks were probably not reported in primary studies. Indeed, the description of the respective theoretical background of digital interventions may not be sufficiently reported in primary studies for it to be coded by reviewers [51]. Second, a focus on theoretical frameworks for development but not for the evaluation of user outcomes suggests that some digital interventions may be developed for profit, while benefits to users remain secondary or unclear. Theoretical frameworks appear to inform mechanisms of action (how digital interventions work) but are also required to define how digital interventions affect user outcomes and contribute to behavior change [25]. Third, the highly heterogeneous terminology used in the field of digital interventions means that the terms *evaluation* or *evaluation frameworks* may not have been explicitly mentioned in primary studies or reviews. Since the term *evaluation* was included in our search syntax, we only identified reviews that specifically referred to evaluation in titles, abstracts, or key words. Thus, more literature on theoretical frameworks in the context of digital interventions likely exists but was not located using our strategy. Indeed, the reviews included in this scoping review cited different primary studies meaning that the overlap in the primary literature among the reviews was very low despite the common topic (digital interventions for physical activity promotion). There were only 4 primary studies [60-63] that were cited in 5 to 6 systematic reviews. Interestingly, all 4 studies are reasonably old (published 2014-2017), given the rapid technological advancement and interest in digital tools to support physical activity. In general, all 4 studies [60-63] compared the physical activity outcomes of digital interventions supported by different digital tools with or without other engagement methods, such as human coaching, reminders, or feedback. The results and implications of these 4 studies can be summarized as follows: (1) physical activity outcomes were evaluated using objective tool data, (2) similar physical activity benefits were evident when using modern digital tools with feedback, such as smartphone apps or activity trackers, to those evident when using traditional tools, such as pedometers, (3) physical activity benefits were higher when digital interventions were combined with human support or feedback, and (4) preference for and acceptance of modern digital tools was high based on feedback from participants and use patterns recorded by the tools. Future research is required to determine the benefits of digital interventions relative to baseline physical activity and to evaluate the effectiveness of complex interventions incorporating digital tools and human coaching for physical activity promotion.

### Evidence Gaps and Ideas for Future Research

Our results suggest that the production of yet another scoping review of primary literature on the topic of evaluation of digital interventions for physical activity promotion (planned as part 2 of this review) may not be necessary and could contribute to research waste. Instead, based on our results of part 1 of our study (this scoping review of reviews) we propose the following topics for future research.

First, more work is needed to identify factors that could improve the effectiveness of digital interventions for physical activity promotion. According to the majority of the included reviews, the identification of such factors could help to increase adherence to digital interventions and contribute to evaluation of efficacy of digital interventions. Digital interventions are typically complex interventions that require several elements for their effectiveness, such as personalization, feedback, engagement with the tool, or human support [64]. The contribution of these elements to the success or failure of digital interventions for physical activity promotion is unclear, primarily because this information was either not coded in reviews or not reported in the primary studies. Furthermore, sociodemographic factors, including age, gender, education, income, and digital health literacy, affect the use of and interest in digital health technologies [1] and could also facilitate or hinder the efficacy of digital interventions. Further research is needed to identify health needs or barriers associated with digital health technology use in low socioeconomic settings [65] to improve the efficacy of digital interventions for physical activity promotion in such populations [66].

Second, evaluation guidelines are required for digital interventions because complex interventions, such as digital interventions, are often insufficiently reported [67]. Until these guidelines are in place, for the description of digital interventions, authors could use already established reporting guidelines, such as the TIDieR Checklist [68], which includes a description of the rationale, theory, or goal of the elements essential to the intervention in item 2. Therefore, adherence to this reporting guideline could improve the inclusion of theoretical frameworks for digital interventions in future studies.

Third, standardized reporting of digital intervention components and outcomes is needed. The key difficulty is that a standardized and universally accepted definition of digital interventions does not exist yet. Generic terms, such as eHealth, mobile health (mHealth), or telehealth typically refer to very different digital health approaches. A recent guideline from the World Health Organization [69] refers to digital interventions as digitally supported interventions delivered via the internet or digital tools with mobile apps. This definition includes the elements of eHealth and mHealth and encompasses the modern digital tools, such as smartphone apps, but also established technologies, such as the internet in general (websites and email), mobile phones able to deliver SMS reminders or wearable sensors able to quantify physical activity. There are two main differences among any of these digital interventions: (1) the level of digital health literacy required to operate or interact with the digital technologies included in the digital intervention, which can be low for noninteractive wearable sensors to high when operating a smartphone app, and (2) the level of engagement and feedback, which can range from a passive use of a website, obtaining reminders via email or SMS to continuous tracing and feedback from smartphone apps or activity trackers. Furthermore, similar to that of nondigital interventions, the development of core physical activity outcome sets is necessary to evaluate digital interventions in different populations [70,71].

Fourth, objective and homogeneous outcome measures should be evaluated for digital interventions using appropriate study designs with long-term follow-up. The potential for digital interventions to improve health has been scarcely realized, partly due to an insufficient knowledge base of guiding principles in the development and evaluation of such interventions [72]. While the gold standard for evaluating a health intervention is conducting a randomized controlled trial, these can take a long time and typically require many participants and extensive financial resources. Long delays to evaluate novel digital interventions in the rapidly evolving field might result in the digital interventions becoming obsolete or nonfunctional by the time the trial is completed [73]. Thus, the evaluation of digital interventions potentially requires new study designs and methods that take the iterative and rapidly evolving nature of such interventions and continuous data collection into account. Furthermore, digital interventions are at the intersection of various fields, such as behavioral, biomedical, and computing sciences. Thus, methods taken from multiple disciplines are required for development and outcome evaluation of digital interventions [74].

Fifth, the reviews included in this scoping review predominantly focused on mixed healthy or clinical samples and predominantly adult populations. Since digital interventions can support health promotion and disease prevention [1], future research should focus on the evaluation of digital interventions or digital tools in healthy populations to promote healthy lifestyle, including physical activity. Furthermore, children and adolescents are important target populations for digital interventions that focus on physical activity promotion because the use of digital technologies contributes to sedentary behavior, especially in early childhood [75]. Therefore, future research should consider potential benefits but also harms of digital technologies and thus, evaluate if digital interventions promote or hinder physical activity in children and adolescents [76].

### Evidence Appraisal

Although this scoping review focused on evaluation strategies rather than outcomes of such evaluations, we performed an appraisal of the 30 systematic reviews included in our study with the AMSTAR2 tool. We found that the overall methodological quality of systematic reviews of digital interventions for physical activity promotion needs improvement, which has already been suggested in the context of other health interventions [77-79]. The overall confidence in the results of systematic reviews of digital interventions for physical activity promotion could be improved by better adherence to established reporting guidelines for systematic reviews and the prospective registration of review protocols. Due to potential financial interests in the field of digital interventions, the sources of funding for primary studies should be documented in systematic reviews. Our results are in line with those of other studies [80-82] that assessed the methodological quality of systematic reviews in telemedicine [80]; digital methods for maximizing participant engagement, participation, and retention in cohort studies [81]; or digital interventions for reducing behavioral risks of cardiovascular disease [82]. These studies [80-82] demonstrated that the majority or all of the included systematic reviews regarding digital interventions had low methodological quality, and thus, the overall confidence in the results of these systematic reviews was considered to be critically low.

### Limitations

There were several limitations in this scoping review. First, the search strategy for literature was conservative due to the inclusion of the term *evaluation* in the syntax. Thus, we did not find reviews of digital interventions for physical activity promotion that omitted the term *evaluation* from their titles, abstracts, or keywords. A manual search for such reviews was beyond the scope of this review. Second, study selection and data coding were difficult due to highly heterogeneous terminology for digital interventions, physical activity outcomes and evaluation. These difficulties contributed to partially superficial coding of data with little detail on specific aspects of evaluation. We also struggled to code the data item *evaluation target* into user outcomes or tool performance. While the evaluation of well-defined user outcomes (ie, promotion of a specific physical activity outcome) was a focus of most reviews, the focus on tool performance was less clear and sometimes included as part of user outcomes (eg, acceptance of the tool). Although any 2 authors coded the data and reached consensus by discussion, a coding manual with more detail could have improved the quality of coding.

### Conclusions

The evaluation of digital interventions is a high priority based on the 40 reviews included in this scoping review. Evaluations of digital interventions, including mobile apps or wearables for physical activity promotion, typically target any user outcomes and rely on objective tool data. While the development of digital interventions appears to be guided by various aspects of behavior change theory, evaluation frameworks are also required to evaluate user outcomes. Behavior change theory may provide useful guidance not only for the development of digital interventions but also for the evaluation of user outcomes in the context of physical activity promotion. Evidence gaps mentioned in most reviews included a need to (1) identify factors that could improve the effectiveness of digital interventions for physical activity promotion, such as personalization, feedback, engagement with the tool, human support, and digital literacy; (2) develop evaluation guidelines; and (3) standardize the reporting of digital intervention components and outcomes. The implementation of evaluation frameworks at the development stage and to assess user outcomes is required to ensure that digital interventions are effective for physical activity promotion.

## Appendix

Multimedia Appendix 1 Additional material.

Multimedia Appendix 2 Search strategy and results.

Multimedia Appendix 3 Overall confidence ratings (using AMSTAR2).

Multimedia Appendix 4 Coded data.

## Abbreviations

AMSTAR2: A Measurement Tool to Assess Systematic Reviews, version 2
mHealth: mobile health
PICOS: Population, Intervention, Comparison, Outcome, Study
PRISMA-ScR: Preferred Reporting Items for Systematic Reviews and Meta-analyses Extension for Scoping Reviews
