# Supplementary material for: Evaluation of Digital Interventions for Physical Activity Promotion: Scoping Review
Source: JMIR Public Health Surveill. 2022 May 23;8(5):e37820. doi: 10.2196/37820 (PMC9171604; doi:10.2196/37820)
Supplement: Multimedia Appendix 1 [file publichealth_v8i5e37820_app1.pdf]

Multimedia Appendix 1

Evaluation of Digital Interventions for Physical Activity Promotion: Scoping Review

De Santis, K. K., Jahnel, T., Matthias, K., Mergenthal, L., Al Khayyal, H., & Zeeb, H.. Evaluation of Digital Interventions for Physical Activity Promotion: Scoping Review. JMIR Public Health Surveill. 2022; doi: 10.2196/37820

Content

Table S1. PRISMA-ScR Checklist. 2
Table S2. List of included and excluded studies. DI: digital intervention; PA: physical activity. 5
Figure S1. Study characteristics of 10 rapid and scoping reviews. 6
Figure S2. Study characteristics of 30 systematic reviews. 7
Textbox S1. Overlap among primary studies cited in 40 reviews. 8
Figure S3. Evaluation strategies addressed in 40 reviews. 9
References 10

Table S1. PRISMA-ScR Checklist.

Section	Item	PRISMA-ScR Checklist Item	Fulfilled / Location
Title	1	Identify the report as a scoping review.	yes
**Abstract**			
Structured summary	2	Provide a structured summary that includes (as applicable): background, objectives, eligibility criteria, sources of evidence, charting methods, results, and conclusions that relate to the review questions and objectives.	yes
**Introduction**			
Rationale	3	Describe the rationale for the review in the context of what is already known. Explain why the review questions/objectives lend themselves to a scoping review approach.	yes
Objectives	4	Provide an explicit statement of the questions and objectives being addressed with reference to their key elements (e.g., population or participants, concepts, and context) or other relevant key elements used to conceptualize the review questions and/or objectives.	yes
**Methods**			
Protocol and registration	5	Indicate whether a review protocol exists; state if and where it can be accessed (e.g., a Web address); and if available, provide registration information, including the registration number.	yes
Eligibility criteria	6	Specify characteristics of the sources of evidence used as eligibility criteria (e.g., years considered, language, and publication status), and provide a rationale.	Textbox 1
Information sources	7	Describe all information sources in the search (e.g., databases with dates of coverage and contact with authors to identify additional sources), as well as the date the most recent search was executed.	yes
Search	8	Present the full electronic search strategy for at least 1 database, including any limits used, such that it could be repeated.	Multimedia Appendix 2
Selection of sources of evidence	9	State the process for selecting sources of evidence (i.e., screening and eligibility) included in the scoping review.	Multimedia Appendix 1, Table S2
Data charting	10	Describe the methods of charting data from	yes

process		the included sources of evidence (e.g., calibrated forms or forms that have been tested by the team before their use, and whether data charting was done independently or in duplicate) and any processes for obtaining and confirming data from investigators.	
Data items	11	List and define all variables for which data were sought and any assumptions and simplifications made.	Textbox 2
Critical appraisal of individual sources of evidence	12	If done, provide a rationale for conducting a critical appraisal of included sources of evidence; describe the methods used and how this information was used in any data synthesis (if appropriate).	yes
Summary measures	13	*Not applicable for scoping reviews*	-
Synthesis of results	14	Describe the methods of handling and summarizing the data that were charted.	yes
Risk of bias across studies	15	*Not applicable for scoping reviews*	-
Additional analyses	16	*Not applicable for scoping reviews*	-
**Results**			
Selection of sources of evidence	17	Give numbers of sources of evidence screened, assessed for eligibility, and included in the review, with reasons for exclusions at each stage, ideally using a flow diagram.	Table 1
Characteristics of sources of evidence	18	For each source of evidence, present characteristics for which data were charted and provide the citations.	Figures S1-S2
Critical appraisal within sources of evidence	19	If done, present data on critical appraisal of included sources of evidence (see item 12).	Figure 3
Results of individual sources of evidence	20	For each included source of evidence, present the relevant data that were charted that relate to the review questions and objectives.	Figure S3
Synthesis of results	21	Summarize and/or present the charting results as they relate to the review questions and objectives.	Figure 2, Figure 4, Textbox S1, Table 2
Risk of bias	22	*Not applicable for scoping reviews*	-

across studies			
Additional analyses	23	*Not applicable for scoping reviews*	-
**Discussion**			
Summary of evidence	24	Summarize the main results (including an overview of concepts, themes, and types of evidence available), link to the review questions and objectives, and consider the relevance to key groups.	yes
Limitations	25	Discuss the limitations of the scoping review process.	yes
Conclusions	26	Provide a general interpretation of the results with respect to the review questions and objectives, as well as potential implications and/or next steps.	yes
**Funding**	27	Describe sources of funding for the included sources of evidence, as well as sources of funding for the scoping review. Describe the role of the funders of the scoping review.	yes

'Yes' means that the item was addressed in the subheading corresponding to section name in the checklist.

Source: Tricco AC, Lillie E, Zarin W, O'Brien KK, Colquhoun H, Levac D, et al. Prisma Extension For Scoping Reviews (Prisma-Scr): Checklist and explanation. Ann Intern Med. 2018;169(7):467-73. doi: 10.7326/m18-0850.

Table S2. List of included and excluded studies. DI: digital intervention; PA: physical activity.

Review type	Studies n	Inclusion or exclusion reason
**Included from electronic and manual searches**	**40**	
Rapid review	1	Inclusion: [1]
Scoping review	9	Inclusion: [2-10]
Systematic review	30	Inclusion: [11-40]
**Reviews from electronic search**	**300**	
**(1) Overview**	**3/300**	
exclude title/abstract	3	Exclusion 2 (no DI for PA): [41-43]
exclude full-text	0	
include	0	
**(2) Rapid review**	**4/300**	
exclude title/abstract	3	Exclusion 2 (no DI for PA): [44-46]
exclude full-text	0	
include	1	Inclusion: [1]
**(3) Scoping review**	**21/300**	
exclude title/abstract	1	Exclusion 1 (non human): [47]
exclude title/abstract	10	Exclusion 2 (no DI for PA): [48-57]
exclude full-text	1	Exclusion 2 (no DI for PA): [58]
include	9	Inclusion: [2-10]
**(4) Narrative review**	**51/300**	
exclude title/abstract	45	Exclusion 2 (no DI for PA): [59-103]
exclude full-text	6	Exclusion 2 (no DI for PA): [104-109]
include	0	
**(5) Systematic review**	**221/300**	
exclude title/abstract	177	Exclusion 2 (no DI for PA): [110-286]
exclude full-text	17	Exclusion 2 (no DI for PA): [287-303]
exclude full-text	1	Exclusion 5 (conference paper): [304]
include	26	Inclusion: [11, 13, 15-27, 29-32, 34-40]
**Reviews from manual search**	**4**	
**Systematic review**		
include	4	Inclusion: [12, 14, 28, 33]

Figure S1. Study characteristics of 10 rapid and scoping reviews.

Citation	Author, year, region (AM: North America, AS: Asia, AUS: Australia, EU: Europe), conflict of interest (NC: no conflict, NR: not reported)	Population: Clinical	Population: Healthy	Population: Any	Population: Minors	Population: Adults	Population: Any age	Intervention: Any digital intervention (DI)	Comparison: Any or none	Outcome: Physical activity promotion	Review type: RR: rapid, ScR: scoping	Primary studies in review	Primary studies: Randomized controlled trials (RCT)	Primary studies: Any designs
1	Morrison 2020 AUS NC	◊				□		telehealth: web-based, mobile apps, SMS, telephone	○	❁	RR	29		●
2	Gluck 2017 AUS NC	◊					□	wearable devices: smartphones, pedometry, accelerometry, GPS	○	❁	ScR	7		●
3	McCallum 2018 EU NR			◊			□	apps or wearables with sensor-based feedback	○	❁	ScR	111		●
4	Aromatario 2019 EU NC			◊			□	mobile health apps: wearable devices, smartphones	○	❁	ScR	22	●	
5	Ghanvatkar 2019 AS NC			◊			□	DI with personalized feedback	○	❁	ScR	49		●
6	Lee 2019 AM NC			◊	□			mHealth intervention: wearable, website, app	○	❁	ScR	16		●
7	Cajita 2020 AM NC			◊			□	wearable activity monitors (wam)	○	❁	ScR	65		●
8	Meinhart 2020 EU NC	◊				□		mobile technologies (mTechs): smartphones, smartwatches, fitness trackers	○	❁	ScR	13	●	
9	Wattanapisit 2020 AS NC			◊		□		eHealth DI: websites, apps	○	❁	ScR	30		●
10	Sporrel 2021 EU NC		◊			□		mobile system: pedometer, SMS, mobile app	○	❁	ScR	29		●

Figure S2. Study characteristics of 30 systematic reviews.

Citation	Author, year, region (AM: North America, AS: Asia, AUS: Australia, EU: Europe), conflict of interest (NC: none, NR: not reported)	Population: Clinical	Population: Healthy	Population: Any	Population: Minors	Population: Adults	Population: Any age	Intervention: Any digital intervention (DI)	Comparison: Any or none	Outcome: Physical activity promotion	Review type: SR: systematic	Primary studies in review	Primary studies: Randomized controlled trials (RCT)	Primary studies: Any designs
11	van den Berg 2007 EU NC			◊		□		internet-based intervention	○	⊛	SR	10		•
12	Fanning 2012 AM NC			◊			□	SMS, mobile software, personal digital assistant	○	⊛	SR	11		•
13	Bort-Roig 2014 EU NC			◊			□	smartphone-based intervention	○	⊛	SR	26		•
14	Bossen 2014 EU NR	◊				□		web-based self-guided interventions that incorporate minimal human support	○	⊛	SR	7	•	
15	Tabak 2015 EU NC			◊			□	mobile games or gamified applications	○	⊛	SR	11		•
16	Ridgers 2016 AUS NC			◊	□			wearable device, wearable activity trackers	○	⊛	SR	5		•
17	Direito 2017 AUS NC		◊				□	mHealth interventions	○	⊛	SR	21	•	
18	Martinez-Garcia 2017 EU NC	◊					□	smartphone	○	⊛	SR	8		•
19	Roberts 2017 EU NC	◊				□		digital health behavior change interventions (DBCI)	○	⊛	SR	15		•
20	Berry 2018 EU NC	◊				□		mobile phone apps, website, online-courses, email-reminders	○	⊛	SR	9		•
21	Cotie 2018 AM NC			◊		□		eHealth interventions: wearable health and movement trackers, websites, smartphone apps, text messaging, emails, voicemail, videogames or tele-health	○	⊛	SR	60		•
22	Griffiths 2018 EU NC	◊					□	interactive DI accessed through any digital platform (computers, smartphones or handheld devices, web-based programmes, wearable technology or apps)	○	⊛	SR	5	•	
23	Haberlin 2018 EU NC	◊					□	eHealth-based intervention delivered by internet or mobile technologies	○	⊛	SR	10		•
24	Yayun 2018 AS NC			◊		□		smartphones or basic mobile phones	○	⊛	SR	8		•
25	Braakhuis 2019 EU NC	◊				□		wearable monitor used for feedback, data collection (frequency, visualization), therapist or coach contact and behavior change theory components	○	⊛	SR	14	•	
26	Brickwood 2019 AUS NC			◊		□		consumer-based/for purchase wearable activity tracker for monitoring/automated real-time feedback via smartphone or web-based platform	○	⊛	SR	28	•	
27	Kim 2019 AS NC		◊			□		smartphone-based apps	○	⊛	SR	5		•
28	Romeo 2019 AUS NC			◊		□		smartphone apps	○	⊛	SR	9	•	
29	Bunting 2020 EU NC	◊				□		DI delivered in electronic format (video games, apps and voice recordings)	○	⊛	SR	5	•	
30	Kwan 2020 AS NC			◊		□		eHealth intervention: any form of electronic devices, internet or other digital technologies	○	⊛	SR	38	•	
31	Laranjo 2020 AUS NC		◊			□		DI with mobile app or activity tracker with automated/continuous self-monitoring and feedback	○	⊛	SR	28	•	
32	Liu 2020 AS NC			◊		□		wearable activity tracker (WAT)-based intervention	○	⊛	SR	10	•	
33	Lynch 2020 AUS NC			◊		□		fitness tracker	○	⊛	SR	21		•
34	Oliveira 2020 AUS NC			◊		□		activity tracker-based DI	○	⊛	SR	23	•	
35	Pradal-Cano 2020 EU NC			◊		□		DI based on mobile apps	○	⊛	SR	14	•	
36	Silva 2020 EU NC			◊		□		DI delivered with mobile app alone or combined with other treatments	○	⊛	SR	11		•
37	Tang 2020 AUS NC		◊			□		DI using wearable trackers with objective feedback alone or with other interventions	○	⊛	SR	12	•	
38	Chan 2021 EU NC	◊				□		exergaming with virtual reality or interactive components with feedback, activity-tracking fitness devices, computerized balance training	○	⊛	SR	9		•
39	He 2021 AS NC			◊	□			smartphone as DI tool with app, SMS or both	○	⊛	SR	9	•	
40	McLaughlin 2021 AUS NC			◊		□		digital, mobile or wireless technologies to achieve health objectives	○	⊛	SR	19		•

Textbox S1. Overlap among primary studies cited in 40 reviews.

10 rapid and scoping reviews

- 371 primary studies included in 10 rapid and scoping reviews.
- 312/371 (84.1%) primary studies published in academic journals.
- 278/312 (89.1%) unique primary studies cited in all reviews.
  - 244/278 (87.8%) studies cited ×1
  - 34/278 (12.2%) studies cited ×2

30 systematic reviews

- 461 primary studies included in 30 systematic reviews.
- 435/461 (94.4%) primary studies published in academic journals.
- 320/435 (73.6%) unique primary studies cited in all reviews.
  - 249/320 (77.8%) studies cited ×1
  - 42/320 (13.1%) studies cited ×2
  - 20/320 (6.2%) studies cited ×3
  - 5/320 (1.6%) studies cited ×4
  - 2/320 (0.6%) studies cited ×5
  - 2/320 (0.6%) studies cited ×6

Figure S3. Evaluation strategies addressed in 40 reviews.

Citation	Author, year	Review type: RR: rapid, ScR: scoping, SR: systematic	Evaluation target: User outcomes	Evaluation target: Tool performance or validation	Evaluation method: Objective tool data	Evaluation method: Other data (self-reports, tests, assessments)	Theory framework (BCT: behaviour change theory)	Theory framework context	Theory framework requirement (tool development vs. outcome evaluation)
1	Morrison 2020	RR	user		objective	other			
2	Gluck 2017	ScR	user	tool	objective	other			outcome
3	McCallum 2018	ScR		tool	objective	other			development, outcome
4	Aromatario 2019	ScR	user			other	BCT, other	development	outcome
5	Ghanvatkar 2019	ScR	user		objective		BCT	development	outcome
6	Lee 2019	ScR	user	tool	objective	other			development, outcome
7	Cajita 2020	ScR	user		objective	other	BCT	development	outcome
8	Meinhart 2020	ScR	user		objective	other	BCT	development	outcome
9	Wattanapisit 2020	ScR	user	tool	objective	other			
10	Sporrel 2021	ScR		tool	objective		BCT, other	development	outcome
11	van den Berg 2007	SR	user			other			outcome
12	Fanning 2012	SR	user	tool	objective				development, outcome
13	Bort-Roig 2014	SR	user	tool	objective		BCT, other	development	
14	Bossen 2014	SR	user			other			
15	Tabak 2015	SR	user	tool	objective	other	BCT	development	outcome
16	Ridgers 2016	SR	user	tool	objective	other			
17	Direito 2017	SR	user	tool	objective		BCT		
18	Martinez-Garcia 2017	SR	user	tool	objective	other			
19	Roberts 2017	SR	user	tool		other	BCT, other	development	outcome
20	Berry 2018	SR	user	tool	objective	other	BCT	development	outcome
21	Cotie 2018	SR	user	tool	objective	other	BCT	development	
22	Griffiths 2018	SR	user	tool	objective	other			
23	Haberlin 2018	SR	user	tool	objective	other	BCT	development	
24	Yayun 2018	SR	user		objective	other			
25	Braakhuis 2019	SR	user		objective		BCT	development	
26	Brickwood 2019	SR	user		objective	other	BCT	development	
27	Kim 2019	SR	user		objective	other	BCT	development	
28	Romeo 2019	SR	user	tool	objective		BCT	development	
29	Bunting 2020	SR	user	tool	objective	other			
30	Kwan 2020	SR	user	tool	objective	other	BCT	development	
31	Laranjo 2020	SR	user		objective		BCT	development	
32	Liu 2020	SR	user	tool	objective		BCT	development	
33	Lynch 2020	SR	user		objective		BCT	development	
34	Oliveira 2020	SR	user	tool	objective				
35	Pradal-Cano 2020	SR	user	tool	objective	other	BCT		
36	Silva 2020	SR	user	tool	objective	other			
37	Tang 2020	SR	user		objective	other			
38	Chan 2021	SR	user			other	BCT	development	
39	He 2021	SR	user	tool	objective				
40	McLaughlin 2021	SR	user		objective	other			

References

1. Morrison KS, Paterson C, Toohey K. The feasibility of exercise interventions delivered via telehealth for people affected by cancer: A rapid review of the literature. Semin Oncol Nurs. 2020;36(6):151092.
2. Gluck S, Chapple LS, Chapman MJ, Iwashyna TJ, Deane AM. A scoping review of use of wearable devices to evaluate outcomes in survivors of critical illness. Crit Care Resusc. 2017;19(3):197-204.
3. McCallum C, Rooksby J, Gray CM. Evaluating the impact of physical activity apps and wearables: Interdisciplinary review. JMIR MHealth and UHealth. 2018;6(3):e58.
4. Aromatario O, Van Hoye A, Vuillemin A, Foucaut A, Crozet C, Pommier J, et al. How do mobile health applications support behaviour changes? A scoping review of mobile health applications relating to physical activity and eating behaviours. Public Health. 2019 Oct;175:8-18.
5. Ghanvatkar S, Kankanhalli A, Rajan V. User models for personalized physical activity interventions: Scoping review. JMIR MHealth and UHealth. 2019;7(1):e11098.
6. Lee AM, Chavez S, Bian J, Thompson LA, Gurka MJ, Williamson VG, et al. Efficacy and effectiveness of mobile health technologies for facilitating physical activity in adolescents: Scoping review. JMIR MHealth and UHealth. 2019;7(2):e11847.
7. Cajita MI, Kline CE, Burke LE, Bigini EG, Imes CC. Feasible but not yet efficacious: A scoping review of wearable activity monitors in interventions targeting physical activity, sedentary behavior, and sleep. Curr Epidemiol Rep. 2020;7(1):25-38. doi: 10.1007/s40471-020-00229-2.
8. Meinhart F, Stutz T, Sareban M, Kulnik ST, Niebauer J. Mobile technologies to promote physical activity during cardiac rehabilitation: A scoping review. Sensors. 2020;21(1):24.
9. Wattanapisit A, Tuangratananon T, Wattanapisit S. Usability and utility of eHealth for physical activity counselling in primary health care: A scoping review. BMC Fam Pract. 2020;21(1). doi: 10.1186/s12875-020-01304-9.
10. Sporrel K, Nibbeling N, Wang S, Ettema D, Simons M. Unraveling mobile health exercise interventions for adults: Scoping review on the implementations and designs of persuasive strategies. JMIR MHealth and UHealth. 2021;9(1):e16282. doi: 10.2196/16282.
11. van den Berg MH, Schoones JW, Vlieland TP. Internet-based physical activity interventions: A systematic review of the literature. JMIR. 2007;9(3):71-86.
12. Fanning J, Mullen SP, McAuley E. Increasing physical activity with mobile devices: a meta-analysis. J Med Internet Res. 2012 Nov 21;14(6):e161. doi: 10.2196/jmir.2171.
13. Bort-Roig J, Gilson N, Puig-Ribera A, Contreras R, Trost S. Measuring and influencing physical activity with smartphone technology: A systematic review. Sports Med. 2014;44(5):671-86. doi: 10.1007/s40279-014-0142-5.
14. Bossen D, Veenhof C, Dekker J, de Bakker D. The effectiveness of self-guided web-based physical activity interventions among patients with a chronic

disease: a systematic review. J Phys Act Health. 2014 Mar;11(3):665-77. doi: 10.1123/jpah.2012-0152.
15. Tabak M, Dekker-van Weering M, van Dijk H, Vollenbroek-Hutten M. Promoting daily physical activity by means of mobile gaming: A review of the state of the art. Games Health J. 2015;4(6):460-9. doi: 10.1089/g4h.2015.0010.
16. Ridgers ND, McNarry MA, Mackintosh KA. Feasibility and effectiveness of using wearable activity trackers in youth: A systematic review. JMIR MHealth and UHealth. 2016;4(4):e129.
17. Direito A, Carraça E, Rawstorn J, Whittaker R, Maddison R, Carraça E. mhealth technologies to influence physical activity and sedentary behaviors: Behavior change techniques, systematic review and meta-analysis of randomized controlled trials. Ann Behav Med. 2017;51(2):226-39. doi: 10.1007/s12160-016-9846-0.
18. Martinez-Garcia MDM, Ruiz-Cardenas JD, Rabinovich RA. Effectiveness of smartphone devices in promoting physical activity and exercise in patients with chronic obstructive pulmonary disease: A systematic review. COPD: J Chron Obstruct Pulmon Dis. 2017;14(5):543-51.
19. Roberts AL, Fisher A, Smith L, Heinrich M, Potts HW. Digital health behaviour change interventions targeting physical activity and diet in cancer survivors: A systematic review and meta-analysis. J Cancer Surviv. 2017 Dec;11(6):704-19.
20. Berry A, McCabe CS, Muir S, Walsh N. Digital behaviour change interventions to facilitate physical activity in osteoarthritis: A systematic review. Phys Ther Rev. 2018;23(3):197-206. doi: 10.1080/10833196.2018.1470747.
21. Cotie LM, Prince SA, Elliott CG, Ziss MC, McDonnell LA, Mullen KA, et al. The effectiveness of eHealth interventions on physical activity and measures of obesity among working-age women: A systematic review and meta-analysis. Obes Rev. 2018;19(10):1340-58.
22. Griffiths AJ, White CM, Thain PK, Bearne LM. The effect of interactive digital interventions on physical activity in people with inflammatory arthritis: A systematic review. Rheumatology International. 2018;38(9):1623-34.
23. Haberlin C, O'Dwyer T, Mockler D, Moran J, O'Donnell DM, Broderick J. The use of eHealth to promote physical activity in cancer survivors: A systematic review. Support Care Cancer. 2018;26(10):3323-36.
24. Yayun S, Jia Q, Di Z, Jun Z. Feasibility and effectiveness of mobile phones in physical activity promotion for adults 50 years and older: A systematic review. Top Geriatr Rehabil. 2018;34(3):213-22. doi: 10.1097/TGR.0000000000000197.
25. Braakhuis HEM, Berger MAM, Bussmann JBJ. Effectiveness of healthcare interventions using objective feedback on physical activity: A systematic review and meta-analysis. J Rehabil Med. 2019;51(3):151-9.
26. Brickwood KJ, Watson G, O'Brien J, Williams AD. Consumer-based wearable activity trackers increase physical activity participation: Systematic review and meta-analysis. JMIR MHealth and UHealth. 2019;7(4):e11819.

27. Kim HN, Seo K. Smartphone-based health program for improving physical activity and tackling obesity for young adults: A systematic review and meta-analysis. Int J Environ Res Public Health. 2019;17(1):18.
28. Romeo A, Edney S, Plotnikoff R, Curtis R, Ryan J, Sanders I, et al. Can smartphone apps increase physical activity? Systematic review and meta-analysis. J Med Internet Res. 2019 Mar 19;21(3):e12053. doi: 10.2196/12053.
29. Bunting JW, Withers TM, Heneghan NR, Greaves CJ. Digital interventions for promoting exercise adherence in chronic musculoskeletal pain: A systematic review and meta-analysis. Physiotherapy. 2020;15:15.
30. Kwan RYC, Salihu D, Lee PH, Tse M, Cheung DSK, Roopsawang I, et al. The effect of e-health interventions promoting physical activity in older people: A systematic review and meta-analysis. Eur Rev Aging Phys Act. 2020;17:7.
31. Laranjo L, Ding D, Heleno B, Kocaballi B, Quiroz JC, Tong HL, et al. Do smartphone applications and activity trackers increase physical activity in adults? Systematic review, meta-analysis and metaregression. Br J Sports Med. 2020;55(8):422-32. doi: 10.1136/bjsports-2020-102892.
32. Liu JY-W, Kor PP-K, Chan CP-Y, Kwan RY-C, Sze-Ki D. The effectiveness of a wearable activity tracker (WAT)-based intervention to improve physical activity levels in sedentary older adults: A systematic review and meta-analysis. Arch Gerontol Geriatr. 2020;91. doi: 10.1016/j.archger.2020.104211.
33. Lynch C, Bird S, Lythgo N, Selva-Raj I. Changing the physical activity behavior of adults with fitness trackers: A systematic review and meta-analysis. Am J Health Promot. 2020 May;34(4):418-30. doi: 10.1177/0890117119895204.
34. Oliveira J, Sherrington C, E RYZ, Franco MR, Tiedemann A. Effect of interventions using physical activity trackers on physical activity in people aged 60 years and over: A systematic review and meta-analysis. Br J Sports Med. 2020;54(20):1188-94.
35. Pradal-Cano L, Lozano-Ruiz C, Pereyra-Rodriguez JJ, Saigi-Rubio F, Bach-Faig A, Esquius L, et al. Using mobile applications to increase physical activity: A systematic review. Int J Environ Res Public Health. 2020;17(21):07.
36. Silva AG, Simoes P, Queiros A, N PR, Rodrigues M. Effectiveness of mobile applications running on smartphones to promote physical activity: A systematic review with meta-analysis. Int J Environ Res Public Health. 2020;17(7):27. doi: 10.3390/ijerph17072251.
37. Tang MSS, Moore K, McGavigan A, Clark RA, Ganesan AN. Effectiveness of wearable trackers on physical activity in healthy adults: Systematic review and meta-analysis of randomized controlled trials. JMIR MHealth and UHealth. 2020;8(7):e15576.
38. Chan C, Sounderajah V, Normahani P, Acharya A, Markar SR, Darzi A, et al. Wearable activity monitors in home based exercise therapy for patients with intermittent claudication: A systematic review. Eur J Vasc Endovasc Surg. 2021;12:12.

39. He Z, Wu H, Yu F, Fu J, Sun S, Huang T, et al. Effects of smartphone-based interventions on physical activity in children and adolescents: Systematic review and meta-analysis. JMIR MHealth and UHealth. 2021;9(2):e22601.
40. McLaughlin M, Delaney T, Hall A, Byaruhanga J, Mackie P, Grady A, et al. Associations between digital health intervention engagement, physical activity, and sedentary behavior: Systematic review and meta-analysis. JMIR. 2021;23(2):e23180.
41. Duncan M, Moschopoulou E, Herrington E, Deane J, Roylance R, Jones L, et al. Review of systematic reviews of non-pharmacological interventions to improve quality of life in cancer survivors. BMJ Open. 2017;7(11):e015860.
42. Eze ND, Mateus C, Cravo Oliveira Hashiguchi T. Telemedicine in the OECD: An umbrella review of clinical and cost-effectiveness, patient experience and implementation. PLoS ONE. 2020;15(8):e0237585. doi: 10.1371/journal.pone.0237585.
43. King AC, Whitt-Glover MC, Marquez DX, Buman MP, Napolitano MA, Jakicic J, et al. Physical activity promotion: Highlights from the 2018 physical activity guidelines advisory committee systematic review. Med Sci Sports Exerc. 2019;51(6):1340-53.
44. Chan CS, Slaughter SE, Jones CA, Ickert C, Wagg AS. Measuring activity performance of older adults using the activpal: A rapid review. Healthcare. 2017;5(4):13.
45. Fischer R, Bortolini T, Karl JA, Zilberberg M, Robinson K, Rabelo A, et al. Rapid review and meta-meta-analysis of self-guided interventions to address anxiety, depression, and stress during COVID-19 social distancing. Front Psychol. 2020;11:563876.
46. Veazie S, Winchell K, Gilbert J, Paynter R, Ivlev I, Eden KB, et al. Rapid evidence review of mobile applications for self-management of diabetes. J Gen Intern Med. 2018;33(7):1167-76. doi: 10.1007/s11606-018-4410-1.
47. Egan S, Brama P, McGrath D. Research trends in equine movement analysis, future opportunities and potential barriers in the digital age: A scoping review from 1978 to 2018. Equine Vet J. 2019;51(6):813-24.
48. Dagenais M, Cheng D, Salbach NM, Brooks D, O'Brien KK. Wireless physical activity monitor use among adults living with HIV: A scoping review. Rehabil Oncol. 2019;37(1):1-28. doi: 10.1097/01.REO.0000000000000153.
49. Black DA, O'Loughlin K, Wilson LA. Climate change and the health of older people in Australia: A scoping review on the role of mobile applications (apps) in ameliorating impact. Australas J Ageing. 2018;37(2):99-106.
50. Kellstedt DK, Spengler JO, Foster M, Lee C, Maddock JE. A scoping review of bikeability assessment methods. J Community Health. 2021;46(1):211-24. doi: 10.1007/s10900-020-00846-4.
51. Petrusevski C, Choo S, Wilson M, MacDermid J, Richardson J. Interventions to address sedentary behaviour for older adults: A scoping review. Disabil Rehabil. 2020:1-12.
52. Wicaksana AL, Hertanti NS, Ferdiana A, Pramono RB. Diabetes management and specific considerations for patients with diabetes during coronavirus

diseases pandemic: A scoping review. Diabetes Metab Syndr. 2020;14(5):1109-20.
53. Kramer LL, Ter Stal S, Mulder BC, de Vet E, van Velsen L. Developing embodied conversational agents for coaching people in a healthy lifestyle: Scoping review. JMIR. 2020;22(2):e14058.
54. Seppen BF, den Boer P, Wiegel J, Ter Wee MM, van der Leeden M, de Vries R, et al. Asynchronous mhealth interventions in rheumatoid arthritis: Systematic scoping review. JMIR MHealth and UHealth. 2020;8(11):e19260.
55. Small SR, Bullock GS, Khalid S, Barker K, Trivella M, Price AJ. Current clinical utilisation of wearable motion sensors for the assessment of outcome following knee arthroplasty: A scoping review. BMJ Open. 2019;9(12):e033832.
56. Taj F, Klein MCA, van Halteren A. Digital health behavior change technology: Bibliometric and scoping review of two decades of research. JMIR MHealth and UHealth. 2019;7(12):e13311.
57. Tully L, Burls A, Sorensen J, El-Moslemany R, O'Malley G. Mobile health for pediatric weight management: Systematic scoping review. JMIR MHealth and UHealth. 2020;8(6):e16214.
58. Baderol Allam FN, Ab Hamid MR, Buhari SS, Md Noor H. Web-based dietary and physical activity intervention programs for patients with hypertension: Scoping review. JMIR. 2021;23(3):e22465.
59. Aida A, Svensson T, Svensson AK, Chung UI, Yamauchi T. ehealth delivery of educational content using selected visual methods to improve health literacy on lifestyle-related diseases: Literature review. JMIR MHealth and UHealth. 2020;8(12):e18316.
60. Andre D, Wolf DL. Recent advances in free-living physical activity monitoring: A review. J Diabetes Sci Technol. 2007;1(5):760-7.
61. Bassett DR, Jr., Toth LP, LaMunion SR, Crouter SE. Step counting: A review of measurement considerations and health-related applications. Sports Med. 2017;47(7):1303-15.
62. Bonomi A, Westerterp K. Advances in physical activity monitoring and lifestyle interventions in obesity: A review. Int J Obes. 2012 Feb;36(2):167-77.
63. Buck HG, Shadmi E, Topaz M, Sockolow PS. An integrative review and theoretical examination of chronic illness mHealth studies using the Middle‐Range Theory of Self‐care of Chronic Illness. Res Nurs Health. 2021;44(1):47-59. doi: 10.1002/nur.22073.
64. Carter H, Araya R, Anjur K, Deng D, Naslund JA. The emergence of digital mental health in low-income and middle-income countries: A review of recent advances and implications for the treatment and prevention of mental disorders. J Psychiatr Res. 2021;133:223-46.
65. Conelea CA, Wellen BC. Tic treatment goes tech: A review of TicHelper.com. Cogn Behav Pract. 2017 Aug;24(3):374-81.

66. Coughlin SS, Whitehead M, Sheats JQ, Mastromonico J, Hardy D, Smith SA. Smartphone applications for promoting healthy diet and nutrition: A literature review. Jacobs Journal of Food and Nutrition. 2015;2(3):021.
67. de Oliveira Gondim ITG, de Souza CCB, Rodrigues MAB, Azevedo IM, de Sales Coriolano M, Lins OG. Portable accelerometers for the evaluation of spatio-temporal gait parameters in people with Parkinson's disease: An integrative review. Arch Gerontol Geriatr. 2020;90:104097.
68. Derksen JWG, Beijer S, Koopman M, Verkooijen HM, van de Poll-Franse LV, May AM. Monitoring potentially modifiable lifestyle factors in cancer survivors: A narrative review on currently available methodologies and innovations for large-scale surveillance. Eur J Cancer. 2018;103:327-40. doi: 10.1016/j.ejca.2018.06.017.
69. Dickman Portz J. A review of web-based chronic disease self-management for older adults. Gerontechnology. 2017;16(1):12-20. doi: 10.4017/gt.2017.16.1.002.00.
70. Dijkstra HP, Ergen E, Holtzhausen L, Beasley I, Alonso JM, Geertsema L, et al. Remote assessment in sport and exercise medicine (SEM): A narrative review and teleSEM solutions for and beyond the COVID-19 pandemic. Br J Sports Med. 2020;54(19):1162-7.
71. Frederix I, Vanhees L, Dendale P, Goetschalckx K. A review of telerehabilitation for cardiac patients. J Telemed Telecare. 2015;21(1):45-53.
72. Garvey C, Singer JP, Bruun AM, Soong A, Rigler J, Hays S. Moving pulmonary rehabilitation into the home: A clinical review. J Cardiopulm Rehabil Prev. 2018;38(1):8-16.
73. Geib RW, Swink PJ, Vorel AJ, Shepard CS, Gurovich AN, Waite GN. The bioengineering of changing lifestyle and wearable technology: A mini review. Biomed Sci Instrum. 2015;51:69-76.
74. Giggins OM, Clay I, Walsh L. Physical activity monitoring in patients with neurological disorders: A review of novel body-worn devices. Digit Biomark. 2017;1(1):14-42.
75. Gray J, O'Malley P. Review: E-health interventions improve blood pressure level and control in hypertension. ACP J Club. 2019;170(12):JC68-JC. doi: 10.7326/ACPJ201906180-068.
76. Houser SH, Joseph R, Puro N, Burke DE. Use of technology in the management of obesity: A literature review. Perspect Health Inf Manag. 2019 Fall2019:1-18.
77. Jung S, Michaud M, Oudre L, Dorveaux E, Gorintin L, Vayatis N, et al. The use of inertial measurement units for the study of free living environment activity assessment: A literature review. Sensors. 2020;20(19):01.
78. Knowles LM, Skeath P, Jia M, Najafi B, Thayer J, Sternberg EM. New and future directions in integrative medicine research methods with a focus on aging populations: A review. Gerontology. 2016;62(4):467-76.
79. Kobe CM, Turcotte LM, Sadak KT. A narrative literature review and environmental scan of self-management education programs for adolescent and young adult survivors of childhood cancer. J Cancer Educ. 2020;35(4):731-5.

80. Lee S, Lindquist R. A review of technology-based interventions to maintain weight loss. Telemed J E Health. 2015;21(3):217-32.
81. Lua PL, Wan Putri Elena WD. The impact of nutrition education interventions on the dietary habits of college students in developed nations: A brief review. Malays J Med Sci. 2012;19(1):4-14.
82. Melanson EL, Jr., Freedson PS. Physical activity assessment: A review of methods. Crit Rev Food Sci Nutr. 1996;36(5):385-96.
83. Melcher J, Hays R, Torous J. Digital phenotyping for mental health of college students: A clinical review. Evid-Based Ment Health. 2020;23(4):161-6.
84. Moral-Munoz JA, Esteban-Moreno B, Herrera-Viedma E, Cobo MJ, Perez IJ. Smartphone applications to perform body balance assessment: A standardized review. J Med Syst. 2018;42(7):119.
85. Nici L, ZuWallack R. They can't bury you while you're still moving: A review of the European Respiratory Society statement on physical activity in chronic obstructive pulmonary disease. Pol Arch Intern Med. 2015;125(10):771-8.
86. Nunes A, Castro SL, Limpo T. A review of mindfulness-based apps for children. Mindfulness. 2020 Sep;11(9):2089-101.
87. Ortega-Martin ME, Lucena-Anton D, Luque-Moreno C, Heredia-Rizo AM, Moral-Munoz JA. [Commercial mobile applications in the therapeutic approach to stroke: Review in main application repositories and scientific evidence]. Rev Esp Salud Publica. 2019;93:12.
88. Peddle-McIntyre CJ, Cavalheri V, Boyle T, McVeigh JA, Jeffery E, Lynch BM, et al. A review of accelerometer-based activity monitoring in cancer survivorship research. Med Sci Sports Exerc. 2018;50(9):1790-801.
89. Pericleous P, van Staa TP. The use of wearable technology to monitor physical activity in patients with COPD: A literature review. Int J Chron Obstruct Pulmon Dis. 2019;14:1317-22.
90. Prabhu NV, Maiya AG, Prabhu NS. Impact of cardiac rehabilitation on functional capacity and physical activity after coronary revascularization: A scientific review. Cardiol Res Pract. 2020:1-9. doi: 10.1155/2020/1236968.
91. Revere D, Dunbar PJ. Review of computer-generated outpatient health behavior interventions: Clinical encounters "in absentia". J Am Med Inform Assoc. 2001;8(1):62-79.
92. Sasaki JE, Sandroff B, Bamman M, Motl RW. Motion sensors in multiple sclerosis: Narrative review and update of applications. Expert Rev Med Devices. 2017;14(11):891-900.
93. Schoeppe S, Alley S, Rebar AL, Hayman M, Bray NA, Van Lippevelde W, et al. Apps to improve diet, physical activity and sedentary behaviour in children and adolescents: a review of quality, features and behaviour change techniques. Int J Behav Nutr Phys Act. 2017;14:1-10. doi: 10.1186/s12966-017-0538-3.
94. Selzler AM, Wald J, Sedeno M, Jourdain T, Janaudis-Ferreira T, Goldstein R, et al. Telehealth pulmonary rehabilitation: A review of the literature and an example of a nationwide initiative to improve the accessibility of pulmonary rehabilitation. Chronic Obstr Pulm Dis. 2018;15(1):41-7.

95. Smith KE, Mason TB, Juarascio A, Schaefer LM, Crosby RD, Engel SG, et al. Moving beyond self‐report data collection in the natural environment: A review of the past and future directions for ambulatory assessment in eating disorders. Int J Eat Disord. 2019;52(10):1157-75. doi: 10.1002/eat.23124.
96. Tedesco S, Barton J, O'Flynn B. A review of activity trackers for senior citizens: Research perspectives, commercial landscape and the role of the insurance industry. Sensors. 2017;17(6):03.
97. Thatipelli S, Arun A, Chung P, Etemadi M, Heller JA, Kwiat D, et al. Review of existing brace adherence monitoring methods to assess adherence. J Prosthet Orthot. 2016;28(4):126-35. doi: 10.1097/JPO.0000000000000106.
98. Tran D-MT, Sojobi A. Review of the scientific literature on young adults related to cardiovascular disease intervention. Asian Pac Isl Nurs J. 2020;5(1):35-46. doi: 10.31372/20200501.1084.
99. van Mechelen DM, van Mechelen W, Verhagen EA. Sports injury prevention in your pocket?! Prevention apps assessed against the available scientific evidence: A review. Br J Sports Med. 2014;48(11):878-82.
100. Williams V, Brown N, Becks A, Pekmezi D, Demark-Wahnefried W. Narrative review of web-based healthy lifestyle interventions for cancer survivors. Annals of Reviews & Research. 2020;5(4).
101. Wu M, Luo J. Wearable technology applications in healthcare: A literature review. Online J Nurs. 2019 Fall2019;23(3):8-1.
102. Yang CC, Hsu YL. A review of accelerometry-based wearable motion detectors for physical activity monitoring. Sensors. 2010;10(8):7772-88.
103. Yeroushalmi S, Maloni H, Costello K, Wallin MT. Telemedicine and multiple sclerosis: A comprehensive literature review. J Telemed Telecare. 2020;26(7-8):400-13.
104. Coughlin SS, Stewart J. Use of consumer wearable devices to promote physical activity: A review of health intervention studies. J Environ Health. 2016;2(6).
105. Coughlin SS, Whitehead M, Sheats JQ, Mastromonico J, Smith S. A review of smartphone applications for promoting physical activity. Jacobs Journal of Community Medicine. 2016;2(1).
106. Ferrer DA, Ellis R. A review of physical activity interventions delivered via Facebook. J Phys Act Health. 2017 Oct;14(10):823-33.
107. Howland C, Wakefield B. Assessing telehealth interventions for physical activity and sedentary behavior self‐management in adults with type 2 diabetes mellitus: An integrative review. Res Nurs Health. 2021;44(1):92-110. doi: 10.1002/nur.22077.
108. Jee H. Review of researches on smartphone applications for physical activity promotion in healthy adults. J Exerc Rehabil. 2017;13(1):3-11.
109. Thilarajah S, Clark RA, Williams G. Wearable sensors and mobile health (mhealth) technologies to assess and promote physical activity in stroke: A narrative review. Brain Impair. 2016 Mar;17(1):34-42.
110. Adamse C, Dekker-Van Weering MGH, van Etten-Jamaludin FS, Stuiver MM, Dekker-Van Weering MG. The effectiveness of exercise-based telemedicine

on pain, physical activity and quality of life in the treatment of chronic pain: A systematic review. J Telemed Telecare. 2018;24(8):511-26. doi: 10.1177/1357633X17716576.

111. Akinosun AS, Polson R, Diaz-Skeete Y, De Kock JH, Carragher L, Leslie S, et al. Digital technology interventions for risk factor modification in patients with cardiovascular disease: Systematic review and meta-analysis. JMIR MHealth and UHealth. 2021;9(3):e21061.
112. Allen JK, Stephens J, Patel A. Technology-assisted weight management interventions: systematic review of clinical trials. Telemed J E Health. 2014;20(12):1103-20.
113. Allet L, Knols RH, Shirato K, de Bruin ED. Wearable systems for monitoring mobility-related activities in chronic disease: A systematic review. Sensors. 2010;10(10):9026-52.
114. Alvarado MM, Hye-Chung K, Coronado KG, Foster MJ, Ortega P, Lawley MA, et al. Barriers to remote health interventions for type 2 diabetes: A systematic review and proposed classification scheme. JMIR. 2017;19(2):1-. doi: 10.2196/jmir.6382.
115. Amatya B, Galea MP, Kesselring J, Khan F. Effectiveness of telerehabilitation interventions in persons with multiple sclerosis: A systematic review. Mult Scler Relat Disord. 2015;4(4):358-69.
116. Angelini S, Alicastro GM, Dionisi S, Di Muzio M. Structure and characteristics of diabetes self-management applications: A systematic review of the literature. CIN. 2019;37(7):340-8. doi: 10.1097/CIN.0000000000000526.
117. Antwi FA, Fazylova N, Garcon M-C, Lopez L, Rubiano R, Slyer JT. Effectiveness of web-based programs on the reduction of childhood obesity in school-aged children: A systematic review. JBI Database System Rev Implement Rep. 2013;11(6):1-44. doi: 10.11124/jbisrir-2013-459.
118. Ayyoubzadeh SM, R. Niakan Kalhori S, Shirkhoda M, Mohammadzadeh N, Esmaeili M. Supporting colorectal cancer survivors using ehealth: A systematic review and framework suggestion. Support Care Cancer. 2020;28(8):3543-55. doi: 10.1007/s00520-020-05372-6.
119. Badawy SM, Kuhns LM. Texting and mobile phone app interventions for improving adherence to preventive behavior in adolescents: A systematic review. JMIR MHealth and UHealth. 2017;5(4):e50.
120. Barakat S, Maguire S, Smith KE, Mason TB, Crosby RD, Touyz S. Evaluating the role of digital intervention design in treatment outcomes and adherence to eTherapy programs for eating disorders: A systematic review and meta - analysis. Int J Eat Disord. 2019;52(10):1077-94. doi: 10.1002/eat.23131.
121. Baskerville R, Ricci-Cabello I, Roberts N, Farmer A. Impact of accelerometer and pedometer use on physical activity and glycaemic control in people with type 2 diabetes: A systematic review and meta-analysis. Diabet Med. 2017;34(5):612-20.
122. Beauchamp UL, Pappot H, Hollander-Mieritz C. The use of wearables in clinical trials during cancer treatment: Systematic review. JMIR MHealth and UHealth. 2020;8(11):e22006.

123. Beishuizen CR, Stephan BC, van Gool WA, Brayne C, Peters RJ, Andrieu S, et al. Web-based interventions targeting cardiovascular risk factors in middle-aged and older people: A systematic review and meta-analysis. JMIR. 2016;18(3):e55.
124. Block VA, Pitsch E, Tahir P, Cree BA, Allen DD, Gelfand JM. Remote physical activity monitoring in neurological disease: A systematic review. PLoS ONE. 2016;11(4):e0154335.
125. Bock C, Jarczok MN, Litaker D. Community-based efforts to promote physical activity: A systematic review of interventions considering mode of delivery, study quality and population subgroups. J Sci Med Sport. 2014;17(3):276-82. doi: 10.1016/j.jsams.2013.04.009.
126. Borde R, Smith JJ, Sutherland R, Nathan N, Lubans DR. Methodological considerations and impact of school-based interventions on objectively measured physical activity in adolescents: A systematic review and meta-analysis. Obes Rev. 2017;18(4):476-90.
127. Bridges CN, Prochnow TM, Wilkins EC, Porter KMP, Meyer MRU. Examining the implementation of play streets: A systematic review of the grey literature. J Public Health Manag Pract. 2020;26(3):E1-E10. doi: 10.1097/PHH.0000000000001015.
128. Brzan PP, Rotman E, Pajnkihar M, Klanjsek P. Mobile applications for control and self management of diabetes: A systematic review. J Med Syst. 2016;40(9):210.
129. Buneviciene I, Mekary RA, Smith TR, Onnela JP, Bunevicius A. Can mHealth interventions improve quality of life of cancer patients? A systematic review and meta-analysis. Crit Rev Oncol Hematol. 2021;157:103123.
130. Burton C, McKinstry B, Tatar AS, Serrano-Blanco A, Pagliari C, Wolters M. Activity monitoring in patients with depression: A systematic review. J Affect Disord. 2013 Feb;145(1):21-8.
131. Butler S, Sculley D, Santos DS, Fellas A, Girones X, Singh-Grewal D, et al. Usability of ehealth and mobile health interventions by young people living with juvenile idiopathic arthritis: Systematic review. JMIR Pediatr Parent. 2020;3(2):e15833.
132. Buyl R, Beogo I, Fobelets M, Deletroz C, Van Landuyt P, Dequanter S, et al. E-health interventions for healthy aging: A systematic review. Syst Rev. 2020;9(1):128.
133. Camomilla V, Bergamini E, Fantozzi S, Vannozzi G. Trends supporting the in-field use of wearable inertial sensors for sport performance evaluation: A systematic review. Sensors. 2018;18(3):15.
134. Champion KE, Parmenter B, McGowan C, Spring B, Wafford QE, Gardner LA, et al. Effectiveness of school-based ehealth interventions to prevent multiple lifestyle risk behaviours among adolescents: A systematic review and meta-analysis. Lancet Digit Health. 2019;1(5):e206-e21.
135. Chan JKY, Klainin-Yobas P, Chi Y, Gan JKE, Chow G, Wu XV. The effectiveness of e-interventions on fall, neuromuscular functions and quality of life in community-dwelling older adults: A systematic review and meta-analysis. Int J Nurs Stud. 2021 Jan;113.

136. Channa A, Popescu N, Ciobanu V. Wearable solutions for patients with Parkinson's disease and neurocognitive disorder: A systematic review. Sensors. 2020;20(9):09.
137. Chen JL, Wilkosz ME. Efficacy of technology-based interventions for obesity prevention in adolescents: A systematic review. Adolesc Health, Med Ther. 2014;5:159-70.
138. Cheung AT, Li WHC, Ho LLK, Ho KY, Chan GCF, Chung JOK. Physical activity for pediatric cancer survivors: A systematic review of randomized controlled trials. J Cancer Surviv. 2021;03:03.
139. Christensen J, Valentiner LS, Petersen RJ, Langberg H. The effect of game-based interventions in rehabilitation of diabetics: A systematic review and meta-analysis. J E Health. 2016 Oct;22(10):789-97.
140. Chung B, Oh E, Song S. Mobile health for breast cancer patients: A systematic review. Asian Oncology Nursing. 2017;17(3):133-42. doi: 10.5388/aon.2017.17.3.133.
141. Clark RA, Conway A, Poulsen V, Keech W, Tirimacco R, Tideman P. Alternative models of cardiac rehabilitation: A systematic review. Eur J Prev Cardiol. 2015;22(1):35-74.
142. Compernolle S, De Cocker K, Lakerveld J, Mackenbach JD, Nijpels G, Oppert J-M, et al. A RE-AIM evaluation of evidence-based multi-level interventions to improve obesity-related behaviours in adults: A systematic review. Int J Behav Nutr Phys Act. 2014;11:1-27. doi: 10.1186/s12966-014-0147-3.
143. Conway A, Schadewaldt V, Clark R, Ski C, Thompson DR, Kynoch K, et al. The effectiveness of non-pharmacological interventions in improving psychological outcomes for heart transplant recipients: A systematic review. Eur J Cardiovasc Nurs. 2014;13(2):108-15.
144. Coorey GM, Neubeck L, Mulley J, Redfern J. Effectiveness, acceptability and usefulness of mobile applications for cardiovascular disease self-management: Systematic review with meta-synthesis of quantitative and qualitative data. Eur J Prev Cardiol. 2018;25(5):505-21.
145. Corti C, Oldrati V, Oprandi MC, Ferrari E, Poggi G, Borgatti R, et al. Remote technology-based training programs for children with acquired brain injury: A systematic review and a meta-analytic exploration. Behav Neurol. 2019;2019:1346987.
146. Cross A, Howlett N, Sheffield D. Social ecological interventions to increase physical activity in children and young people living with and beyond cancer: A systematic review. Psychol Health. 2020;35(12):1477-96.
147. Daly LM, Horey D, Middleton PF, Boyle FM, Flenady V. The effect of mobile app interventions on influencing healthy maternal behavior and improving perinatal health outcomes: Systematic review. JMIR MHealth and UHealth. 2018;6(8):e10012.
148. Darling KE, Sato AF. Systematic review and meta-analysis examining the effectiveness of mobile health technologies in using self-monitoring for pediatric weight management. Child Obes. 2017;13(5):347-55.

149. Davoudi A, Manini TM, Bihorac A, Rashidi P. Role of wearable accelerometer devices in delirium studies: A systematic review. Crit Care Explor. 2019;1(9):e0027.
150. de Araujo AVL, Neiva JFO, Monteiro CBM, Magalhaes FH. Efficacy of virtual reality rehabilitation after spinal cord injury: A systematic review. BioMed Res Int. 2019;2019:7106951.
151. de Vries HJ, Kooiman TJM, van Ittersum MW, van Brussel M, de Groot M. Do activity monitors increase physical activity in adults with overweight or obesity? A systematic review and meta-analysis. Obesity. 2016;24(10):2078-91. doi: 10.1002/oby.21619.
152. Delva S, Waligora Mendez KJ, Cajita M, Koirala B, Rongzi S, Wongvibulsin S, et al. Efficacy of mobile health for self-management of cardiometabolic risk factors: A theory-guided systematic review. J Cardiovasc Nurs. 2021;36(1):34-55. doi: 10.1097/JCN.0000000000000659.
153. Dogan E, Sander C, Wagner X, Hegerl U, Kohls E. Smartphone-based monitoring of objective and subjective data in affective disorders: Where are we and where are we going? Systematic review. JMIR. 2017 Jul;19(7):223-40.
154. Duan Y, Shang B, Liang W, Du G, Yang M, Rhodes RE. Effects of ehealth-based multiple health behavior change interventions on physical activity, healthy diet, and weight in people with noncommunicable diseases: Systematic review and meta-analysis. JMIR. 2021;23(2):e23786.
155. Edwards D, Noyes J, Lowes L, Haf Spencer L, Gregory JW. An ongoing struggle: A mixed-method systematic review of interventions, barriers and facilitators to achieving optimal self-care by children and young people with type 1 diabetes in educational settings. BMC Pediatr. 2014;14:228.
156. Elaheebocus SMRA, Weal M, Morrison L, Yardley L. Peer-based social media features in behavior change interventions: Systematic review. JMIR. 2018;20(2):23. doi: 10.2196/jmir.8342.
157. Fawcett E, Van Velthoven MH, Meinert E. Long-term weight management using wearable technology in overweight and obese adults: Systematic review. JMIR MHealth and UHealth. 2020;8(3):e13461.
158. Federici S, Meloni F, Bracalenti M, De Filippis ML. The effectiveness of powered, active lower limb exoskeletons in neurorehabilitation: A systematic review. Neurorehabilitation. 2015;37(3):321-40.
159. Feehan LM, Geldman J, Sayre EC, Park C, Ezzat AM, Yoo JY, et al. Accuracy of fitbit devices: Systematic review and narrative syntheses of quantitative data. JMIR MHealth and UHealth. 2018;6(8):e10527.
160. Flores Mateo G, Granado-Font E, Ferre-Grau C, Montana-Carreras X. Mobile phone apps to promote weight loss and increase physical activity: A systematic review and meta-analysis. JMIR. 2015;17(11):e253.
161. Gandhi S, Chen S, Hong L, Sun K, Gong E, Li C, et al. Effect of mobile health interventions on the secondary prevention of cardiovascular disease: Systematic review and meta-analysis. Canadian Journal of Cardiology. 2017;33(2):219-31.

162. Georgiou K, Larentzakis AV, Khamis NN, Alsuhaibani GI, Alaska YA, Giallafos EJ. Can wearable devices accurately measure heart rate variability? A systematic review. Folia Medica. 2018;60(1):7-20.
163. Goode A, Hall K, Batch B, Huffman K, Hastings S, Allen K, et al. The impact of interventions that integrate accelerometers on physical activity and weight loss: A systematic review. Ann Behav Med. 2017;51(1):79-93. doi: 10.1007/s12160-016-9829-1.
164. Goode A, Lawler S, Brakenridge C, Reeves M, Eakin E, Goode AD, et al. Telephone, print, and Web-based interventions for physical activity, diet, and weight control among cancer survivors: A systematic review. J Cancer Surviv. 2015;9(4):660-82. doi: 10.1007/s11764-015-0442-2.
165. Goode AD, Reeves MM, Eakin EG. Telephone-delivered interventions for physical activity and dietary behavior change: An updated systematic review. Am J Prev Med. 2012 Jan;42(1):81-8.
166. Gordt K, Gerhardy T, Schwenk M, Najafi B. Effects of wearable sensor-based balance and gait training on balance, gait, and functional performance in healthy and patient populations: A systematic review and meta-analysis of randomized controlled trials. Gerontology. 2017;64(1):74-89. doi: 10.1159/000481454.
167. Graña Possamai C, Ravaud P, Ghosn L, Tran V-T. Use of wearable biometric monitoring devices to measure outcomes in randomized clinical trials: A methodological systematic review. BMC Med. 2020;18(1):N.PAG-N.PAG. doi: 10.1186/s12916-020-01773-w.
168. Guay C, Auger C, Demers L, Mortenson W, Miller WC, Gelinas-Bronsard D, et al. Components and outcomes of internet-based interventions for caregivers of older adults: Systematic review. JMIR. 2017 Sep;19(9):e313.
169. Guest E, Costa B, Williamson H, Meyrick J, Halliwell E, Harcourt D. The effectiveness of interventions aiming to promote positive body image in adults: A systematic review. Body Image. 2019;30:10-25. doi: 10.1016/j.bodyim.2019.04.002.
170. Haghayegh S, Khoshnevis S, Smolensky MH, Diller KR, Castriotta RJ. Accuracy of wristband Fitbit models in assessing sleep: Systematic review and meta-analysis. JMIR. 2019 Nov;21(11):e16273.
171. Hall KS, Hyde ET, Bassett DR, Carlson SA, Carnethon MR, Ekelund U, et al. Systematic review of the prospective association of daily step counts with risk of mortality, cardiovascular disease, and dysglycemia. Int J Behav Nutr Phys Act. 2020;17(1):78.
172. Halldorsdottir H, Thoroddsen A, Ingadottir B. Impact of technology-based patient education on modifiable cardiovascular risk factors of people with coronary heart disease: A systematic review. Patient Educ Couns. 2020 Oct;103(10):2018-28.
173. Hannan AL, Harders MP, Hing W, Climstein M, Coombes JS, Furness J. Impact of wearable physical activity monitoring devices with exercise prescription or advice in the maintenance phase of cardiac rehabilitation: Systematic review and meta-analysis. BMC Sports Sci Med Rehabil. 2019;11:14.

174. Hartmann-Boyce J, Johns DJ, Jebb SA, Summerbell C, Aveyard P, Behavioural Weight Management Review Group. Behavioural weight management programmes for adults assessed by trials conducted in everyday contexts: Systematic review and meta-analysis. Obes Rev. 2014;15(11):920-32.
175. Henriksen A, Johansson J, Hartvigsen G, Grimsgaard S, Hopstock L. Measuring physical activity using triaxial wrist worn polar activity trackers: A systematic review. Int J Exerc Sci. 2020;13(4):438-54.
176. Heydarian H, Adam M, Burrows T, Collins C, Rollo ME. Assessing eating behaviour using upper limb mounted motion sensors: A systematic review. Nutrients. 2019;11(5):24.
177. Hosseiniravandi M, Kahlaee AH, Karim H, Ghamkhar L, Safdari R. Home-based telerehabilitation software systems for remote supervising: A systematic review. Int J Technol Assess Health Care. 2020;36(2):113-25.
178. Hou SI, Charlery SA, Roberson K. Systematic literature review of Internet interventions across health behaviors. Health Psychol Behav Med. 2014;2(1):455-81.
179. Howarth A, Quesada J, Silva J, Judycki S, Mills PR. The impact of digital health interventions on health-related outcomes in the workplace: A systematic review. Digit Health. 2018;4:2055207618770861.
180. Hu Y. Health communication research in the digital age: A systematic review. J Commun Healthc. 2015;8(4):260-88. doi: 10.1080/17538068.2015.1107308.
181. Huang JW, Lin YY, Wu NY. The effectiveness of telemedicine on body mass index: A systematic review and meta-analysis. J Telemed Telecare. 2019;25(7):389-401.
182. Huang K, Liu W, He D, Huang B, Xiao D, Peng Y, et al. Telehealth interventions versus center-based cardiac rehabilitation of coronary artery disease: A systematic review and meta-analysis. Eur J Prev Cardiol. 2015;22(8):959-71.
183. Hubble RP, Naughton GA, Silburn PA, Cole MH. Wearable sensor use for assessing standing balance and walking stability in people with Parkinson's disease: A systematic review. PLoS ONE. 2015;10(4):e0123705.
184. Huguet A, Miller A, Kisely S, Rao S, Saadat N, McGrath PJ. A systematic review and meta-analysis on the efficacy of Internet-delivered behavioral activation. J Affect Disord. 2018 Aug;235:27-38.
185. Hussain T, Smith P, Yee LM. Mobile phone-based behavioral interventions in pregnancy to promote maternal and fetal health in high-income countries: Systematic review. JMIR MHealth and UHealth. 2020;8(5):e15111.
186. Hwang R, Bruning J, Morris N, Mandrusiak A, Russell T. A systematic review of the effects of telerehabilitation in patients with cardiopulmonary diseases. J Cardiopulm Rehabil Prev. 2015;35(6):380-9.
187. Irvine A, Drew P, Bower P, Brooks H, Gellatly J, Armitage CJ, et al. Are there interactional differences between telephone and face-to-face psychological therapy? A systematic review of comparative studies. J Affect Disord. 2020 Mar;265:120-31.
188. Jacob CM, Hardy-Johnson PL, Inskip HM, Morris T, Parsons CM, Barrett M, et al. A systematic review and meta-analysis of school-based interventions with

health education to reduce body mass index in adolescents aged 10 to 19 years. Int J Behav Nutr Phys Act. 2021;18(1):1-22. doi: 10.1186/s12966-020-01065-9.
189. James P, Morgant R, Merviel P, Saraux A, Giroux-Metges MA, Guillodo Y, et al. How to promote physical activity during pregnancy : A systematic review. J Gynecol Obstet Hum Reprod. 2020;49(9):101864.
190. Jimenez-Moreno AC, Newman J, Charman SJ, Catt M, Trenell MI, Gorman GS, et al. Measuring habitual physical activity in neuromuscular disorders: A systematic review. J Neuromuscul Dis. 2017;4(1):25-52.
191. Jo A, Coronel BD, Coakes CE, Mainous AG, 3rd. Is there a benefit to patients using wearable devices such as fitbit or health apps on mobiles? A systematic review. Am J Med. 2019;132(12):1394-400.e1.
192. Job JR, Fjeldsoe BS, Eakin EG, Reeves MM. Effectiveness of extended contact interventions for weight management delivered via text messaging: A systematic review and meta-analysis. Obes Rev. 2018;19(4):538-49.
193. Johansson D, Malmgren K, Alt Murphy M. Wearable sensors for clinical applications in epilepsy, parkinson's disease, and stroke: A mixed-methods systematic review. J Neurol. 2018;265(8):1740-52.
194. Johansson T, Wild C. Telerehabilitation in stroke care-a systematic review. J Telemed Telecare. 2011;17(1):1-6.
195. Johnson EC, Helen Cross J, Reilly C. Physical activity in people with epilepsy: A systematic review. Epilepsia. 2020;61(6):1062-81.
196. Kaakinen P, Kyngas H, Kaariainen M. Technology-based counseling in the management of weight and lifestyles of obese or overweight children and adolescents: A descriptive systematic literature review. Inform Health Soc Care. 2018 Apr;43(2):126-41.
197. Kamei T, Kanamori T, Yamamoto Y, Edirippulige S. The use of wearable devices in chronic disease management to enhance adherence and improve telehealth outcomes: A systematic review and meta-analysis. J Telemed Telecare. 2020:1357633X20937573.
198. Kebapcı A, Ozkaynak M, Lareau SC. Effects of ehealth-based interventions on adherence to components of cardiac rehabilitation: A systematic review. J Cardiovasc Nurs. 2020;35(1):74-85. doi: 10.1097/JCN.0000000000000619.
199. Khan F, Amatya B, Kesselring J, Galea MP. Telerehabilitation for persons with multiple sclerosis. A Cochrane review. Eur J Phys Rehabil Med. 2015;51(3):311-25.
200. Kim M, Kim C, Kim E, Choi M. Effectiveness of mobile health-based exercise interventions for patients with peripheral artery disease: Systematic review and meta-analysis. JMIR MHealth and UHealth. 2021;9(2):e24080.
201. Kiss N, Baguley BJ, Ball K, Daly RM, Fraser SF, Granger CL, et al. Technology-supported self-guided nutrition and physical activity interventions for adults with cancer: Systematic review. JMIR MHealth and UHealth. 2019;7(2):e12281.
202. Kobsar D, Charlton JM, Tse CTF, Esculier JF, Graffos A, Krowchuk NM, et al. Validity and reliability of wearable inertial sensors in healthy adult walking: A systematic review and meta-analysis. J Neuroeng Rehabil. 2020;17(1):62.

203. Krishna S, Boren SA, Balas EA. Healthcare via cell phones: A systematic review. Telemed J E Health. 2009;15(3):231-40.
204. Kuo C-C, Su Y-J, Lin C-C. A systematic review and meta-analysis: Effectiveness of internet empowerment-based self-management interventions on adults with metabolic diseases. J Adv Nurs. 2018 Aug;74(8):1787-802.
205. Lee J, Piao M, Byun A, Kim J. A systematic review and meta-analysis of intervention for pediatric obesity using mobile technology. Stud Health Technol Inform. 2016;225:491-4.
206. Lee M, Lee H, Kim Y, Kim J, Cho M, Jang J, et al. Mobile app-based health promotion programs: A systematic review of the literature. Int J Environ Res Public Health. 2018;15(12):13.
207. Li J, Theng Y-L, Foo S. Game-based digital interventions for depression therapy: A systematic review and meta-analysis. Cyberpsychol Behav Soc Netw. 2014 Aug;17(8):519-27.
208. Liu P, Li G, Jiang S, Liu Y, Leng M, Zhao J, et al. The effect of smart homes on older adults with chronic conditions: A systematic review and meta-analysis. Geriatric Nursing. 2019;40(5):522-30.
209. Lopes de Sousa PM. Effectiveness of e-health intervention programs in obese adolescents: Systematic review of literature. Pensar Enfermagem. 2014;18(1):27-39.
210. Lundell S, Holmner A, Rehn B, Nyberg A, Wadell K. Telehealthcare in COPD: A systematic review and meta-analysis on physical outcomes and dyspnea. Respir Med. 2015;109(1):11-26.
211. Macadam P, Cronin J, Neville J, Diewald S. Quantification of the validity and reliability of sprint performance metrics computed using inertial sensors: A systematic review. Gait Posture. 2019;73:26-38.
212. Macadam P, Cronin J, Simperingham K. The effects of wearable resistance training on metabolic, kinematic and kinetic variables during walking, running, sprint running and jumping: A systematic review. Sports Med. 2017;47(5):887-906. doi: 10.1007/s40279-016-0622-x.
213. Machado GC, Pinheiro MB, Lee H, Ahmed OH, Hendrick P, Williams C, et al. Smartphone apps for the self-management of low back pain: A systematic review. Best Pract Res Clin Rheumatol. 2016;30(6):1098-109.
214. Marin TS, Kourbelis C, Foote J, Newman P, Brown A, Daniel M, et al. Examining adherence to activity monitoring devices to improve physical activity in adults with cardiovascular disease: A systematic review. Eur J Prev Cardiol. 2019;26(4):382-97.
215. Marinho DA, Neiva HP, Morais JE. The use of wearable sensors in human movement analysis in non-swimming aquatic activities: A systematic review. Int J Environ Res Public Health. 2019;16(24):12.
216. McDonough DJ, Su X, Gao Z. Health wearable devices for weight and bmi reduction in individuals with overweight/obesity and chronic comorbidities: Systematic review and network meta-analysis. Br J Sports Med. 2021;17:17.
217. McMahon J, Thompson DR, Pascoe MC, Brazil K, Ski CF. eHealth interventions for reducing cardiovascular disease risk in men: A systematic review and meta-analysis. Prev Med. 2021;145:106402.

218. Mertens L, Braeken M, Bogaerts A. Effect of lifestyle coaching including telemonitoring and telecoaching on gestational weight gain and postnatal weight loss: A systematic review. Telemed J E Health. 2019;25(10):889-901.
219. Milne-Ives M, Lam C, De Cock C, Van Velthoven MH, Meinert E. Mobile apps for health behavior change in physical activity, diet, drug and alcohol use, and mental health: Systematic review. JMIR MHealth and UHealth. 2020;8(3):e17046.
220. Mitchell MS, Orstad SL, Biswas A, Oh PI, Jay M, Pakosh MT, et al. Financial incentives for physical activity in adults: Systematic review and meta-analysis. Br J Sports Med. 2020;54(21):1259-68.
221. Morgan C, Rolinski M, McNaney R, Jones B, Rochester L, Maetzler W, et al. Systematic review looking at the use of technology to measure free-living symptom and activity outcomes in Parkinson's disease in the home or a home-like environment. J Parkinsons Dis. 2020;10(2):429-54.
222. Najm A, Gossec L, Weill C, Benoist D, Berenbaum F, Nikiphorou E. Mobile health apps for self-management of rheumatic and musculoskeletal diseases: Systematic literature review. JMIR MHealth and UHealth. 2019;7(11):e14730.
223. Newby K, Teah G, Cooke R, Li X, Brown K, Salisbury-Finch B, et al. Do automated digital health behaviour change interventions have a positive effect on self-efficacy? A systematic review and meta-analysis. Health Psychol Rev. 2020 Jan;15(1):140-58.
224. Nuss K, Moore K, Nelson T, Li K. Effects of motivational interviewing and wearable fitness trackers on motivation and physical activity: A systematic review. Am J Health Promot. 2021;35(2):226-35.
225. O'Reilly M, Caulfield B, Ward T, Johnston W, Doherty C. Wearable inertial sensor systems for lower limb exercise detection and evaluation: A systematic review. Sports Med. 2018;48(5):1221-46.
226. Oftedal S, Bell KL, Mitchell LE, Davies PS, Ware RS, Boyd RN. A systematic review of the clinimetric properties of habitual physical activity measures in young children with a motor disability. Int J Pediatr. 2012;2012:976425.
227. Oikonomidi T, Vivot A, Tran VT, Riveros C, Robin E, Ravaud P. A methodologic systematic review of mobile health behavior change randomized trials. Am J Prev Med. 2019;57(6):836-43.
228. Oliveira CB, Franco MR, Maher CG, Ferreira PH, Morelhão PK, Damato TM, et al. Physical activity–based interventions using electronic feedback may be ineffective in reducing pain and disability in patients with chronic musculoskeletal pain: A systematic review with meta-analysis. Arch Phys Med Rehabil. 2018;99(9):1900-12. doi: 10.1016/j.apmr.2017.10.013.
229. Palma S, Keilani M, Hasenoehrl T, Crevenna R. Impact of supportive therapy modalities on heart rate variability in cancer patients – a systematic review. Disabil Rehabil. 2020;42(1):36-43. doi: 10.1080/09638288.2018.1514664.
230. Palmer M, Sutherland J, Barnard S, Wynne A, Rezel E, Doel A, et al. The effectiveness of smoking cessation, physical activity/diet and alcohol reduction interventions delivered by mobile phones for the prevention of

non-communicable diseases: A systematic review of randomised controlled trials. PLoS ONE. 2018;13(1):e0189801.
231. Paramastri R, Pratama SA, Ho DKN, Purnamasari SD, Mohammed AZ, Galvin CJ, et al. Use of mobile applications to improve nutrition behaviour: A systematic review. Comput Methods Programs Biomed. 2020;192:105459.
232. Pennington M, Visram S, Donaldson C, White M, Lhussier M, Deane K, et al. Cost-effectiveness of health-related lifestyle advice delivered by peer or lay advisors: Synthesis of evidence from a systematic review. Cost Eff Resour Alloc. 2013;11(1):30.
233. Pfaeffli Dale L, Dobson R, Whittaker R, Maddison R. The effectiveness of mobile-health behaviour change interventions for cardiovascular disease self-management: A systematic review. Eur J Prev Cardiol. 2016;23(8):801-17.
234. Powell L, Parker J, Martyn St-James M, Mawson S. The effectiveness of lower-limb wearable technology for improving activity and participation in adult stroke survivors: A systematic review. JMIR. 2016;18(10):e259.
235. Puigdomenech Puig E, Robles N, Saigi-Rubio F, Zamora A, Moharra M, Paluzie G, et al. Assessment of the efficacy, safety, and effectiveness of weight control and obesity management mobile health interventions: Systematic review. JMIR MHealth and UHealth. 2019;7(10):e12612.
236. Rawstorn JC, Gant N, Direito A, Beckmann C, Maddison R. Telehealth exercise-based cardiac rehabilitation: A systematic review and meta-analysis. Heart. 2016;102(15):1183-92.
237. Reeder B, David A. Health at hand: A systematic review of smart watch uses for health and wellness. J Biomed Inform. 2016;63:269-76. doi: 10.1016/j.jbi.2016.09.001.
238. Rhodes A, Smith AD, Chadwick P, Croker H, Llewellyn CH. Exclusively digital health interventions targeting diet, physical activity, and weight gain in pregnant women: Systematic review and meta-analysis. JMIR MHealth and UHealth. 2020;8(7):e18255.
239. Rintala A, Paivarinne V, Hakala S, Paltamaa J, Heinonen A, Karvanen J, et al. Effectiveness of technology-based distance physical rehabilitation interventions for improving physical functioning in stroke: A systematic review and meta-analysis of randomized controlled trials. Arch Phys Med Rehabil. 2019;100(7):1339-58.
240. Robinson A, Husband AK, Slight RD, Slight SP. Digital technology to support lifestyle and health behaviour changes in surgical patients: Systematic review. BJS Open. 2021;5(2):05.
241. Robinson A, Oksuz U, Slight R, Slight S, Husband A. Digital and mobile technologies to promote physical health behavior change and provide psychological support for patients undergoing elective surgery: Meta-ethnography and systematic review. JMIR MHealth and UHealth. 2020;8(12):e19237.
242. Rogers MAM, Lemmen K, Kramer R, Mann J, Chopra V. Internet-delivered health interventions that work: Systematic review of meta-analyses and

evaluation of website availability. JMIR. 2017;19(3):1-. doi: 10.2196/jmir.7111.
243. Sahin C, Courtney KL, Naylor PJ, R ER. Tailored mobile text messaging interventions targeting type 2 diabetes self-management: A systematic review and a meta-analysis. Digit Health. 2019;5:2055207619845279.
244. Samuel SR, Gandhi AR, Kumar KV, Saxena PP. Pedometer-based exercise interventions for patients with breast cancer receiving chemotherapy - a systematic review. Indian J Palliat Care. 2020;26(1):105-9.
245. Sanyal C, Stolee P, Juzwishin D, Husereau D. Economic evaluations of eHealth technologies: A systematic review. PLoS ONE. 2018;13(6):e0198112.
246. Schafer AGM, Zalpour C, von Piekartz H, Hall TM, Paelke V. The efficacy of electronic health-supported home exercise interventions for patients with osteoarthritis of the knee: Systematic review. JMIR. 2018;20(4):e152.
247. Schaffer K, Panneerselvam N, Loh KP, Herrmann R, Kleckner IR, Dunne RF, et al. Systematic review of randomized controlled trials of exercise interventions using digital activity trackers in patients with cancer. J Natl Compr Canc Netw. 2019;17(1):57-63.
248. Schembre SM, Liao Y, Robertson MC, Dunton GF, Kerr J, Haffey ME, et al. Just-in-time feedback in diet and physical activity interventions: Systematic review and practical design framework. JMIR. 2018 Mar;20(3):e106.
249. Sequi-Dominguez I, Alvarez-Bueno C, Martinez-Vizcaino V, Fernandez-Rodriguez R, Del Saz Lara A, Cavero-Redondo I. Effectiveness of mobile health interventions promoting physical activity and lifestyle interventions to reduce cardiovascular risk among individuals with metabolic syndrome: Systematic review and meta-analysis. JMIR. 2020;22(8):e17790.
250. Shaw G, Whelan ME, Armitage LC, Roberts N, Farmer AJ. Are COPD self-management mobile applications effective? A systematic review and meta-analysis. NPJ Prim Care Respir Med. 2020;30(1):11.
251. Shukla H, Nair S, Thakker D. Role of telerehabilitation in patients following total knee arthroplasty: Evidence from a systematic literature review and meta-analysis. J Telemed Telecare. 2017 Feb;23(2):339-46.
252. Sica M, Tedesco S, Crowe C, Kenny L, Moore K, Timmons S, et al. Continuous home monitoring of Parkinson's disease using inertial sensors: A systematic review. PLoS ONE. 2021;16(2):e0246528.
253. Silfee VJ, Haughton CF, Jake-Schoffman DE, Lopez-Cepero A, May CN, Sreedhara M, et al. Objective measurement of physical activity outcomes in lifestyle interventions among adults: A systematic review. Prev Med Rep. 2018;11:74-80.
254. Smith N, Liu S. A systematic review of the dose-response relationship between usage and outcomes of online physical activity weight-loss interventions. Internet Interv. 2020;22:100344.
255. Spaulding EM, Marvel FA, Piasecki RJ, Martin SS, Allen JK. User engagement with smartphone apps and cardiovascular disease risk factor outcomes: Systematic review. JMIR Cardio. 2021;5(1):e18834.

256. Stellefson M, Chaney B, Barry AE, Chavarria E, Tennant B, Walsh-Childers K, et al. Web 2.0 chronic disease self management for older adults: A systematic review. JMIR. 2013 Feb;15(2):166-79.
257. Storm FA, Cesareo A, Reni G, Biffi E. Wearable inertial sensors to assess gait during the 6-minute walk test: A systematic review. Sensors. 2020;20(9):06.
258. Su JJ, Yu DSF, Paguio JT. Effect of eHealth cardiac rehabilitation on health outcomes of coronary heart disease patients: A systematic review and meta‐analysis. J Adv Nurs. 2020;76(3):754-72. doi: 10.1111/jan.14272.
259. Su MC, Lin CL, Tsao LI. [The efficacy of e-health management on weight control in adolescents: a systematic review]. Hu Li Za Zhi. 2014;61(1):74-84.
260. Tan MKH, Wong JKL, Bakrania K, Abdullahi Y, Harling L, Casula R, et al. Can activity monitors predict outcomes in patients with heart failure? A systematic review. Eur Heart J. 2019;5(1):11-21.
261. Thomas Craig KJ, Morgan LC, Chen CH, Michie S, Fusco N, Snowdon JL, et al. Systematic review of context-aware digital behavior change interventions to improve health. Transl Behav Med. 2020;21:21.
262. Thyregod M, Bodtger U. Coherence between self-reported and objectively measured physical activity in patients with chronic obstructive lung disease: a systematic review. Int J Chron Obstruct Pulmon Dis. 2016;11:2931-8.
263. Tonga E, Srikesavan C, Williamson E, Lamb SE. Components, design and effectiveness of digital physical rehabilitation interventions for older people: A systematic review. J Telemed Telecare. 2020:1357633X20927587.
264. Trifan A, Oliveira M, Oliveira JL. Passive sensing of health outcomes through smartphones: Systematic review of current solutions and possible limitations. JMIR MHealth and UHealth. 2019;7(8):e12649.
265. Tsang K, Hiremath SV, Crytzer TM, Dicianno BE, Ding D. Validity of activity monitors in wheelchair users: A systematic review. J Rehabil Res Dev. 2016;53(6):641-58.
266. Turan Kavradim S, Ozer Z, Boz I. Effectiveness of telehealth interventions as a part of secondary prevention in coronary artery disease: A systematic review and meta-analysis. Scand J Caring Sci. 2020 Sep;34(3):585-603.
267. Turner T, Spruijt-Metz D, Wen C, Hingle M. Prevention and treatment of pediatric obesity using mobile and wireless technologies: A systematic review. Pediatr Obes. 2015 Dec;10(6):403-9.
268. Unal E, Giakoumidakis K, Khan E, Patelarou E. Mobile phone text messaging for improving secondary prevention in cardiovascular diseases: A systematic review. Heart Lung. 2018;47(4):351-9. doi: 10.1016/j.hrtlng.2018.05.009.
269. Vaes AW, Cheung A, Atakhorrami M, Groenen MT, Amft O, Franssen FM, et al. Effect of 'activity monitor-based' counseling on physical activity and health-related outcomes in patients with chronic diseases: A systematic review and meta-analysis. Ann Med. 2013;45(5-6):397-412.
270. Van Remoortel H, Giavedoni S, Raste Y, Burtin C, Louvaris Z, Gimeno-Santos E, et al. Validity of activity monitors in health and chronic disease: A systematic review. Int J Behav Nutr Phys Act. 2012;9:84.

271. Van Rhoon L, Byrne M, Morrissey E, Murphy J, McSharry J. A systematic review of the behaviour change techniques and digital features in technology-driven type 2 diabetes prevention interventions. Digit Health. 2020;6:2055207620914427.
272. Vergani L, Marton G, Pizzoli SFM, Monzani D, Mazzocco K, Pravettoni G. Training cognitive functions using mobile apps in breast cancer patients: Systematic review. JMIR MHealth and UHealth. 2019;7(3):e10855.
273. Vergauwen K, Huijnen IPJ, Depuydt A, Van Regenmortel J, Meeus M. Measuring the physical activity level and pattern in daily life in persons with chronic fatigue syndrome/myalgic encephalomyelitis: A systematic review. Phys Ther Rev. 2017;22(1/2):23-33. doi: 10.1080/10833196.2017.1300624.
274. Verrotti A, Penta L, Zenzeri L, Agostinelli S, De Feo P. Childhood obesity: prevention and strategies of intervention. A systematic review of school-based interventions in primary schools. J Endocrinol Investig. 2014;37(12):1155-64.
275. Versluis A, Verkuil B, Spinhoven P, van der Ploeg MM, Brosschot JF. Changing mental health and positive psychological well-being using ecological momentary interventions: A systematic review and meta-analysis. JMIR. 2016;18(6):152-76. doi: 10.2196/jmir.5642.
276. Walker R, Bennett C, Blumfield M, Gwini S, Ma J, Wang F, et al. Attenuating pregnancy weight gain-what works and why: A systematic review and meta-analysis. Nutrients. 2018;10(7):22.
277. Wang E, Abrahamson K, Liu PJ, Ahmed A. Can mobile technology improve weight loss in overweight adults? A systematic review. West J Nurs Res. 2020;42(9):747-59. doi: 10.1177/0193945919888224.
278. Weber H, Barr C, Gough C, van den Berg M. How commercially available virtual reality-based interventions are delivered and reported in gait, posture, and balance rehabilitation: A systematic review. Phys Ther. 2020;100(10):1805-15.
279. Wickersham A, Petrides PM, Williamson V, Leightley D. Efficacy of mobile application interventions for the treatment of post-traumatic stress disorder: A systematic review. Digit Health. 2019;5:2055207619842986.
280. Wongvibulsin S, Habeos EE, Huynh PP, Xun H, Shan R, Porosnicu Rodriguez KA, et al. Digital health interventions for cardiac rehabilitation: Systematic literature review. JMIR. 2021;23(2):e18773.
281. Wu C, Li Y, Chen J. Hybrid versus traditional cardiac rehabilitation models: A systematic review and meta-analysis. Kardiol Pol. 2018;76(12):1717-24.
282. Wu X, Guo X, Zhang Z. The efficacy of mobile phone apps for lifestyle modification in diabetes: Systematic review and meta-analysis. JMIR MHealth and UHealth. 2019;7(1):e12297.
283. Xu H, Long H. The effect of smartphone app-based interventions for patients with hypertension: Systematic review and meta-analysis. JMIR MHealth and UHealth. 2020;8(10):e21759.
284. Yang J, Yang H, Wang Z, Wang X, Wang Y, Yu X, et al. Self‐management among type 2 diabetes patients via the WeChat application: A systematic

review and meta - analysis. J Clin Pharm Ther. 2021;46(1):4-16. doi: 10.1111/jcpt.13264.
285. Yen HY, Chiu HL. The effectiveness of wearable technologies as physical activity interventions in weight control: A systematic review and meta-analysis of randomized controlled trials. Obes Rev. 2019;20(10):1485-93.
286. Young CL, Dawson S, Berk M, O'Neil A, Jacka FN, Trapani K, et al. Efficacy of online lifestyle interventions targeting lifestyle behaviour change in depressed populations: A systematic review. Aust N Z J Psychiatry. 2018;52(9):834-46. doi: 10.1177/0004867418788659.
287. Larsen RT, Christensen J, Juhl CB, Andersen HB, Langberg H. Physical activity monitors to enhance amount of physical activity in older adults - a systematic review and meta-analysis. Eur Rev Aging Phys Act. 2019;16:7.
288. Matthews J, Win K, Oinas-Kukkonen H, Freeman M. Persuasive technology in mobile applications promoting physical activity: A systematic review. J Med Syst. 2016;40(3):1-13. doi: 10.1007/s10916-015-0425-x.
289. Duff OM, Walsh DMJ, Furlong BA, O'Connor NE, Moran KA, Woods CB. Behavior change techniques in physical activity ehealth interventions for people with cardiovascular disease: Systematic review. JMIR. 2017;19(8):1-. doi: 10.2196/jmir.7782.
290. Hakala S, Rintala A, Immonen J, Karvanen J, Heinonen A, Sjogren T. Effectiveness of physical activity promoting technology-based distance interventions compared to usual care. Systematic review, meta-analysis and meta-regression. Eur J Phys Rehabil Med. 2017;53(6):953-67.
291. Jahangiry L, Farhangi MA, Shab-Bidar S, Rezaei F, Pashaei T. Web-based physical activity interventions: A systematic review and meta-analysis of randomized controlled trials. Public Health. 2017;152:36-46.
292. Rose T, Barker M, Jacob CM, Morrison L, Lawrence W, Strommer S, et al. A systematic review of digital interventions for improving the diet and physical activity behaviors of adolescents. J Adolesc Health. 2017 Dec;61(6):669-77.
293. Rintala A, Hakala S, Paltamaa J, Heinonen A, Karvanen J, Sjogren T. Effectiveness of technology-based distance physical rehabilitation interventions on physical activity and walking in multiple sclerosis: A systematic review and meta-analysis of randomized controlled trials. Disabil Rehabil. 2018;40(4):373-87.
294. Tong HL, Laranjo L. The use of social features in mobile health interventions to promote physical activity: A systematic review. NPJ Digit Med. 2018;1:43.
295. Bohm B, Karwiese SD, Bohm H, Oberhoffer R. Effects of mobile health including wearable activity trackers to increase physical activity outcomes among healthy children and adolescents: Systematic review. JMIR MHealth and UHealth. 2019;7(4):e8298.
296. Buckingham SA, Williams AJ, Morrissey K, Price L, Harrison J. Mobile health interventions to promote physical activity and reduce sedentary behaviour in the workplace: A systematic review. Digit Health. 2019;5:2055207619839883.

297. Prince SA, Elliott CG, Scott K, Visintini S, Reed JL. Device-measured physical activity, sedentary behaviour and cardiometabolic health and fitness across occupational groups: A systematic review and meta-analysis. Int J Behav Nutr Phys Act. 2019;16(1):30.
298. Shin Y, Kim SK, Lee M. Mobile phone interventions to improve adolescents' physical health: A systematic review and meta‐analysis. Public Health Nurs. 2019;36(6):787-99. doi: 10.1111/phn.12655.
299. Stockwell S, Schofield P, Fisher A, Firth J, Jackson SE, Stubbs B, et al. Digital behavior change interventions to promote physical activity and/or reduce sedentary behavior in older adults: A systematic review and meta-analysis. Exp Gerontol. 2019;120:68-87.
300. Chaudhry UAR, Wahlich C, Fortescue R, Cook DG, Knightly R, Harris T. The effects of step-count monitoring interventions on physical activity: Systematic review and meta-analysis of community-based randomised controlled trials in adults. Int J Behav Nutr Phys Act. 2020;17(1):1-16. doi: 10.1186/s12966-020-01020-8.
301. Davis A, Sweigart R, Ellis R. A systematic review of tailored mHealth interventions for physical activity promotion among adults. Transl Behav Med. 2020;10(5):1221-32.
302. Franssen WM, Franssen GH, Spaas J, Solmi F, Eijnde BO. Can consumer wearable activity tracker-based interventions improve physical activity and cardiometabolic health in patients with chronic diseases? A systematic review and meta-analysis of randomised controlled trials. Int J Behav Nutr Phys Act. 2020 May;17:57.
303. Jin D, Halvari H, Maehle N, Olafsen AH. Self-tracking behaviour in physical activity: A systematic review of drivers and outcomes of fitness tracking. Behav Inf Technol. 2020 Aug. doi: 10.1080/0144929X.2020.1801840.
304. Blackwood J, Huang MH. The use of telehealth interventions to improve physical activity in adult cancer survivors: A systematic review. Rehabil Oncol. 2018;36(1):E9-E. doi: 10.1097/01.REO.0000000000000098.
